# Modelling and Experimental Study of Power Losses in Toothed Wheels

**DOI:** 10.3390/s23125541

**Published:** 2023-06-13

**Authors:** Guglielmo Giannetti, Enrico Meli, Andrea Rindi

**Affiliations:** Department of Industrial Engineering, University of Florence, 50139 Florence, Italy

**Keywords:** rolling bearing, gearbox, power loss evaluation, lubrication system, multibody modeling, toothed wheels

## Abstract

In recent decades, the request for more efficient performances in the aeronautical sector moved researchers to pay particular attention to all the related mechanisms and systems, especially with respect to the saving of power. In this context, the bearing modeling and design, as well as gear coupling, play a fundamental role. Moreover, the need for low power losses also concerns the study and the implementation of advanced lubrication systems, especially for high peripheral speed. With the previous aims, this paper presents a new validated model for toothed gears, added to a bearing model; with the link of these different submodels, the whole model describes the system’s dynamic behavior, taking into account the different kinds of power losses (windage losses, fluid dynamic losses, etc.) generated by the mechanical system parts (especially rolling bearings and gears). As the bearing model, the proposed model is characterized by high numerical efficiency and allows the investigation of different rolling bearings and gears with different lubrication conditions and frictions. A comparison between the experimental and simulated results is also presented in this paper. The analysis of the results is encouraging and shows a good agreement between experiments and model simulations, with particular attention to the power losses in the bearing and gears.

## 1. Introduction

Recently, gears and gearboxes have been rediscovered in important industrial sectors, such as aircraft gas turbines and rocket motors. This trend reversal was due to the features of the gears: high mechanical resistance, high reliability and low power consumption. Moreover, due to the different machine layouts thanks to the gear introduction, it is possible to save weight, always a fundamental aspect in the aeronautical sector. In this context, the authors studied an aeronautical gearbox in different operative conditions, adding the gear models to a previous rotodynamic model, with bearings already validated [1]. Due to the extensive diffusion of mechanical systems with rolling bearings and gears, it is important to also investigate their functionality in aeronautical applications. The authors studied the power losses of an entire rotor-dynamic system with no external resistant torque or load because the aim of the paper is to identify all the different sources of power losses. An external resistant torque or load applied on one or both the shafts might make complex the study and the characterization of the power losses independent of the load because they might be negligible compared to the power losses dependent on the load. Especially in the gears, friction emerges extensively during the meshing phase, consequently resulting in mechanical power losses. The mechanical power losses due to different friction and fluid dynamic effects are converted into heat (thermal power losses) that must be dissipated by the lubrication system. Meanwhile, the heat will affect the mechanical interaction and dynamic behavior of the gears and even induce rapid failure, especially when excessive temperature growth occurs. The dynamical and thermal analysis of the gears under friction during this simultaneous process has been of great scientific interest. The paper is focused on the study of the gear contact dynamic behavior. Moreover, this study continues to develop the model presented in the paper “Modeling and experimental study of power losses in a rolling bearing” [1]; in that paper, the tests were realized without the gears, while in the current paper the gears were added on the test rig. In particular, the following paper is based on the rotor-bearings model developed in the paper of Giannetti [1], but it contains also the validation of the rotor-bearings-gear model, adding the studies and the considerations related to the gears. In the model presented, the gears are studied with an energetic point of view, finding which types of power losses are involved in the toothed wheels. To aim a complete research of the power losses related to the gears, the mechanical power losses (due to the friction and fluid dynamic) and the thermal power losses (due to heat dissipation) were considered. Obviously, similar to the previous paper, it is essential to obtain state-of-the-art results.

The main focus of the work proposed answer to the following question: the proposed model for toothed gears and bearing modeling contribute to more efficient performances in aeronautics due to the low time consumption and the good agreement between numerical and experimental data. In this way, the project time of a prototype of a test rig or of a mechanical system might be reduced and the results, meaning the experimental data of the machine manufactured, would already be predicted.

An in-depth study of the state of the art has already been completed by the authors about the rolling bearings for the validation of the mechanical and thermal behavior of rotor-dynamic systems with this type of bearing [1]. The following paragraph presents the theoretical basis of spur gears and the object of the experimental tests. The gears are usually used in conditions where it is important to maintain a constant ratio between the linked shafts: for this reason, it is fundamental to know the dynamic behavior and the contact between gears. The most typical way to reproduce the gear dynamic and the related contact between gears is through models with lumped parameters, stiffness and dampers [2,3,4]. Regarding the contact between tooth gears, a lot of aspects influence the dynamic interaction between gears: non-linear behavior of tooth gears, backlash between tooth gears (important in particular during the motion reversal), geometric parameters of the gears, etc. These aspects are considered by Ebrahimi [5]: their model defined the forces and the torque during the gear’s contact using lumped stiffness and dampers on the tooth gears, depending on the interpenetration and on the relativity velocity in the contact zone.

Other important researchers deepened the behavior of the gear coupling [2,3,4]. The first Bartelmus model [4] considered the torsional vibration, using the two gears with stiffness and damping parameters. Moreover, the model considered the engine torque, the shaft’s stiffness torques, engine and load inertia moments, damping torque coupling and the relative damping coefficient, the stiffness and damping forces between teeth and finally the stiffness shaft. After the previous model described above, Bartelmus also introduced lateral vibration to study the multistage gearbox behavior [4].

Another important contribution to the knowledge of gears coupling behavior was given by Friswell [6]. In their model, he considered the torsional vibration and the gearbox to be mathematically represented by four rotational inertia (engine, load and the two gears) and two torsional stiffness (engine shaft and load shaft). Obviously, the relation between the gear displacements and gear angular velocity is given by the gear ratio. By the Friswell motion equation, the inertia displacements (two gears and two shafts) are obtainable by the external torque agents on inertia and by the instantaneous force exchanged between the gears, along the common tangent.

As already mentioned, to reproduce the gearbox’s dynamic behavior, it is fundamental to know all the loss contributions. One of the most important of these contributions is the windage losses [7,8,9,10]. The windage losses are usually the bigger loss contribution for big gears, high speed and low load. In the previous papers cited, the load windage is studied for isolated spur gears. In particular, Dawson [7] studied the influence of angular velocity, dimension, geometry and protection for a single gear in air. Protection of the gear from the fluid is the most significant method to reduce windage losses. Lord [11] extrapolated three empirical equations to quantify the windage losses for gears with modules ranging from 1 to 5 mm, considering the air density with the ideal gas equation. The windage losses equation and correlation considered in this paper are reported in Massini’s PhD thesis [12].

The pumping losses are another important part of the gear losses. Usually, the pumping losses are higher with oil lubricant, but also with air and dry contact. In fact, the lubricant present between the gear teeth is similar to that in a volumetric pump. In some research, the pumping losses were considered using coefficients dependent on equivalent density (air and oil). Furthermore, for this topic this paper considers the equation and correlation presented in Massini’s PhD thesis [12] and their other published studies [13,14].

In addition to pumping and windage losses, there is one more loss component, the losses due to the lubrication of the gears. Seetharaman and Kahraman [15] studied the gears’ bath lubrication depending on the oil level. The drawback of bath lubricant is the high power losses. For this reason, the lubricant with oil jet was studied by Massini and Fondelli [12,16,17]: in these studies, the oil jet lubricant losses on the gears was studied using the momentum contribution. There are a lot of mechanical applications where the oil jet is positioned along the tangent of the gear’s primitive diameters.

Depending on the angular speed and load, the oil jet can be at the start of the engagement (into-mesh), at the end (out-of-mesh) or both. With oil jet tangential spacing disposal, it is possible to obtain an easier mechanical solution, but this is not the better solution in terms of cooling the root gear [18]. According to Massini and Fondelli, it is possible to obtain a better cooling of the gears with the radial direction of the oil jet, near the end of the engagement: this method requires a lower oil pressure to distribute the lubricant on the tooth gear surfaces, also on the root gears. Tangential and into-mesh oil jet is the lubrication solution with lower gear cooling because the oil has little time before engagement to remove heat, in addition to the higher pumping losses to expel oil from the teeth contact zone. Instead, tangential and into-mesh oil jet is beneficial when lubrication aspects are more important compared to the cooling (very low and very high angular velocity). For example, the most common solution for high-speed gears is to use the tangential oil jet into-mesh to obtain better lubricant and lower pumping losses (with lower oil mass flow), with radial oil jet to increase cooling. Tangential and out-of-mesh oil jet is the lubrication solution used when the tangential and into-mesh oil jet involves high pumping losses and lower cooling (medium-high speed angular velocity). This solution is beneficial with respect to the radial oil jet when the gears coupling does not need of high cooling. Finally, the radial oil jet is very important to guarantee the heat removal needed and to completely cover the surface of the teeth of the gears with oil in case of high angular speed.

Furthermore, the mechanical losses were studied in the literature: Diab and Velex [19,20] realized a method to predict the power losses due to the tooth friction in gears. They concentrated their effort on studying the sliding losses during the contact between the tooth gears. In particular, they studied how the specimen surface texture influences the sliding losses. Furthermore, in other works, they investigated power losses in high-speed gears due to the sum of sliding and rolling frictions between the gear teeth, the effects of the lubrication process, the pumping of a gas-lubricant mixture during meshing and the windage losses.

Similar research was conducted by Benedict and Kelley [21]: they validated a model to predict the instantaneous coefficients of gear tooth friction in the function of the load, velocity, sliding velocity and oil viscosity. Regarding the oil behavior and the mechanical power losses in the toothed wheels, Li and Kahraman [22] developed a prediction model for a pair of spur gears using a transient elastohydrodynamic lubrication model. Furthermore, Magalhães [23] and Michlin [24] worked on the toothed gear power losses: especially the first one was studied, and it was shown that the temperature influences the behavior of the toothed gears to achieve a lower energy consumption and to reduce the risk of failures. In the second study, the experiments concentrated on describing the power losses due to both the rolling and sliding friction.

In the current state of the art, a similar approach to the studies of the bearings and rotor gears is not present, especially regarding the subdivisions of the different types of power losses (mechanical and thermal losses) and in terms of the temperature distributions in the complete rotor system composed of bearings, rotor and gears. Usually, the different power losses are studied separately, without a link to each other. In this paper, the authors had the goal of linking the different types of power losses together, paying particular attention to the adding aspects of the work presented: the toothed wheels’ power losses. As anticipated, starting from the validated bearings-rotor model [1], this study adds the meshing of the toothed wheels to the model, especially studying it in terms of mechanical and thermal power losses.

The proposed model is able to predict the trend of the main physical variables of a complex system in important applications, such as aeronautical. This is possible thanks to the balance between accuracy and numerical efficiency [25]. Some submodels compose the complete proposed model, entirely developed using *Matlab-Simulink R2021b* software: the gear model, which calculates the relationship between the kinematics parameters and the force due to the gear’s contact, is integrated with the rotor-bearing models [1]. Moreover, the simulated results were compared to the experimental data to validate the model, and they show a good agreement. The effects of power losses (resistant torque and bearing and oil temperatures) are well matched by the simulated results during all the phases of the experimental tests. The modeling and experimental test of the proposed aeronautical gearbox were carried out thanks to a collaboration between the *MDM Lab* (Dynamic Modelling and Mechatronics Laboratory) and *THT Lab* (Technology for High-Temperature Laboratory) of the University of Florence. The THT Lab designed a test rig with gears coupling to represent an aeronautical gearbox, while the MDM Lab developed the numerical model to predict the system’s physical behavior.

## 2. Experimental Setup

**Figure 1:**
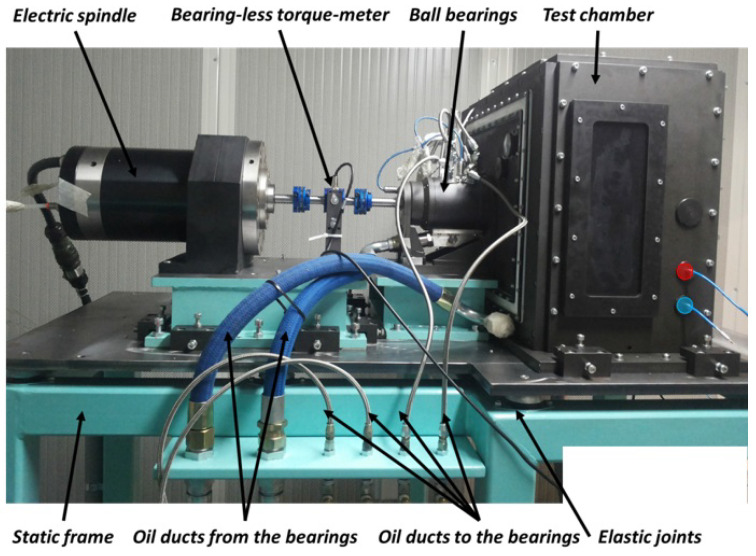
View of the test rig.

Figure 1 shows the test rig object of the studies.

**Figure 2:**
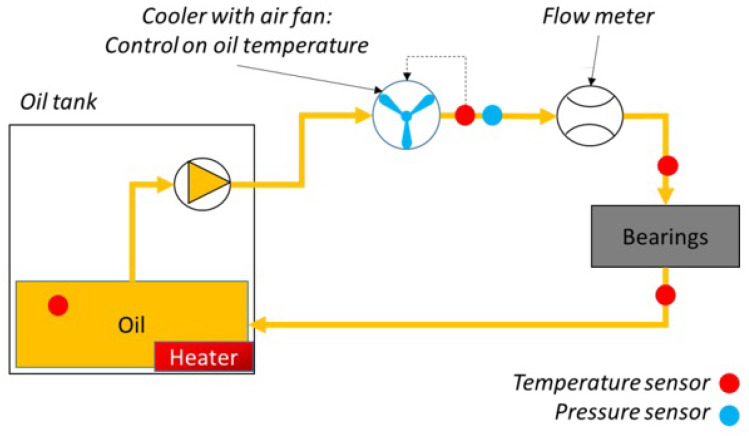
Oil control unit.

The electric motor (maximum speed of 15,000 RPM and maximum torque of 30 Nm) rotates the driving shaft, and both shafts are supported with a pair of angular ball bearings mounted in an “O” configuration and lubricated with oil jet. The oil lubricant ISO 46 is provided with a specific mass flow-rate ${\dot{m}}_{oil}$ and temperature $T_{in}$ by a dedicated oil control unit (Figure 2).

**Figure 3:**
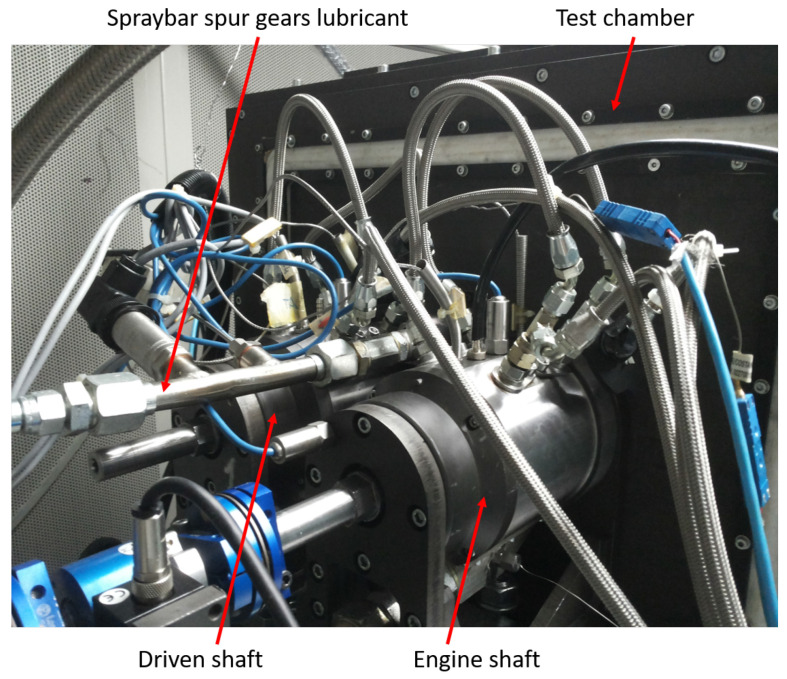
Test ring shafts, test chamber and spraybar.

The coupling between the two shafts is realized with two equal spur gears, so the ratio is equal to “1”. The mechanical characteristics of the two gears are shown in Table 1, while in Figure 3 are presented the main components of the test rig.

Moreover, on both gears, a section to balance torsional and bending vibrations is present. At half of the gear width, which has a diameter equal to 114 mm (inside than the root diameter), there are 24 threaded holes. These holes are used to tighten little concentrated mass to balance gears and shafts. Different combinations of concentrated masses (quantities and positions) were tested for both gears, and the combinations with lower levels of vibrations were chosen.

K-type thermocouples measure the external ring temperatures of both bearings ($T_{oe}$), while other T-type thermocouples check the inlet and outlet oil ducts ($T_{oil}$).

The two gears are in an isolated box, where a vacuum pump reduces the air pressure (the test chamber in Figure 1). Thus, the test rig permits the reproduction of different levels of air pressure in the test chamber to study the windage losses on the gears. The reduction of the test chamber pressure compared to the atmospheric pressure can reach up to 60 kPa. In this way, it is possible to reduce the windage losses, increasing the sealing losses that allowed the separation of the test chamber and the rolling bearings chambers, and it is possible to evaluate the energetic convenience of this proposed solution.

**Figure 4:**
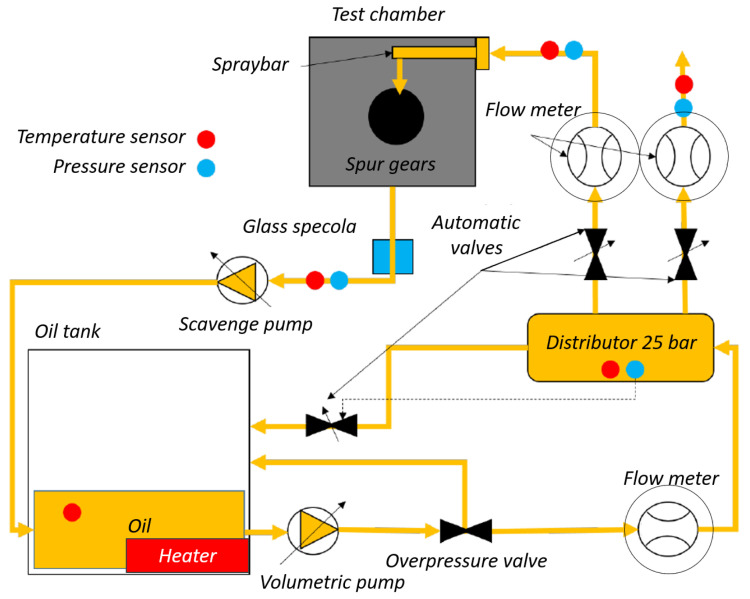
Oil control unit gears.

**Figure 5:**
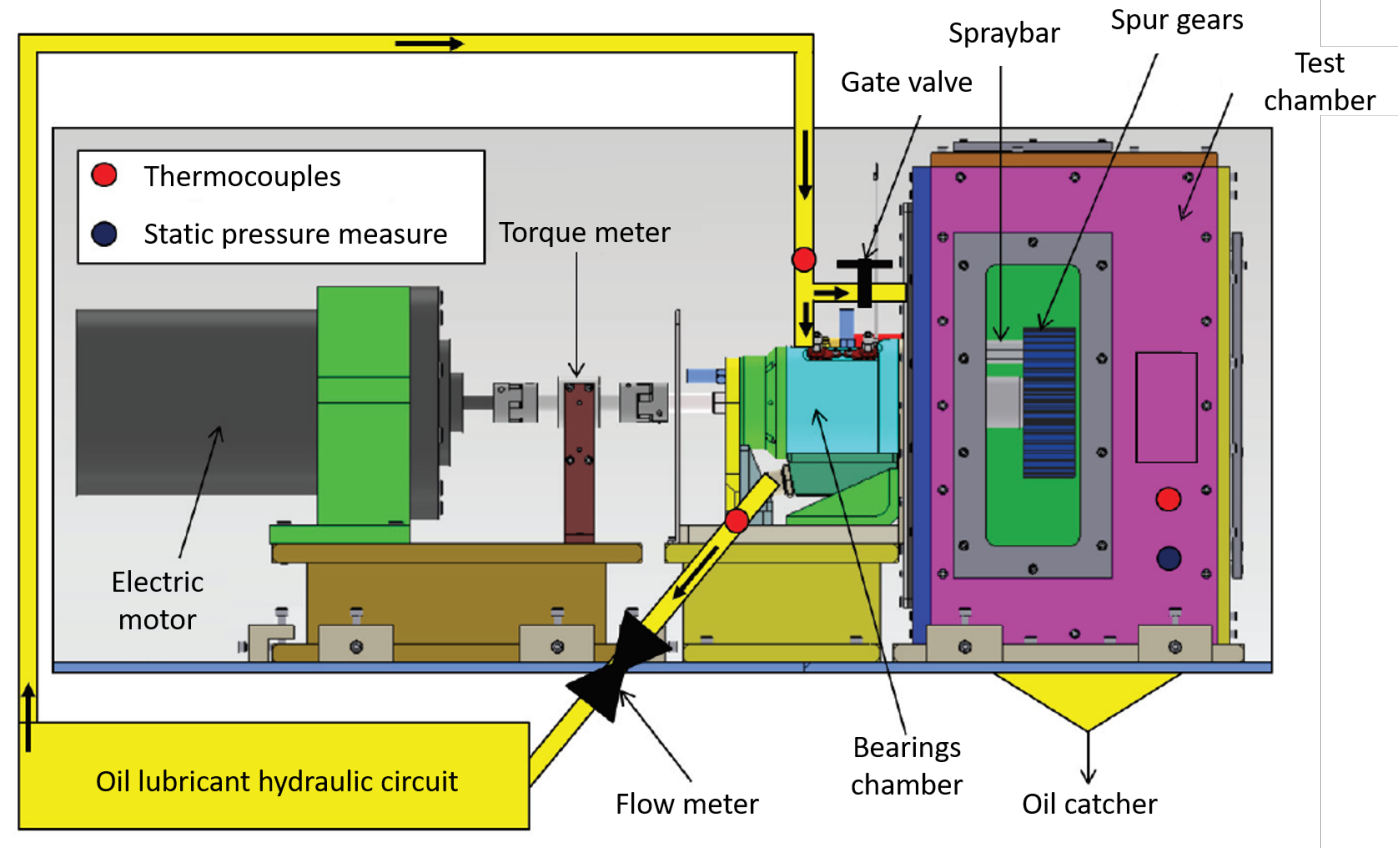
Oil control test ring, bearings and gears.

With respect to the lubricant system, in the test rig, there are different hydraulic circuits to lubricate the gears, separated by the hydraulic circuit to lubricate the rolling bearings. The gears’ lubricant system is represented in Figure 4, while Figure 5 represents the link between the two different oil hydraulic circuits.

The lubricant oil is guaranteed by the spray bar: it is possible to vary the angular timing of the spray bar to lubricate the gears in different ways. The spray bar realized radial lubrication, with four holes (each diameter 1 mm) along the rotational axis. All the measurement data have been acquired using a developed Labview® routine.

The resistant torque measure $M_{b}$ was carried out with a bearingless rotary torque transducer and with an encoder. The pressure in the test chamber is detected with a pressure transducer, linked to a pressure absolute transducer, to measure the pressure difference. All the data from the experimental tests carried out have been acquired using a Labview® process developed for the purpose of the tests.

## 3. Modeling Description

**Figure 6:**
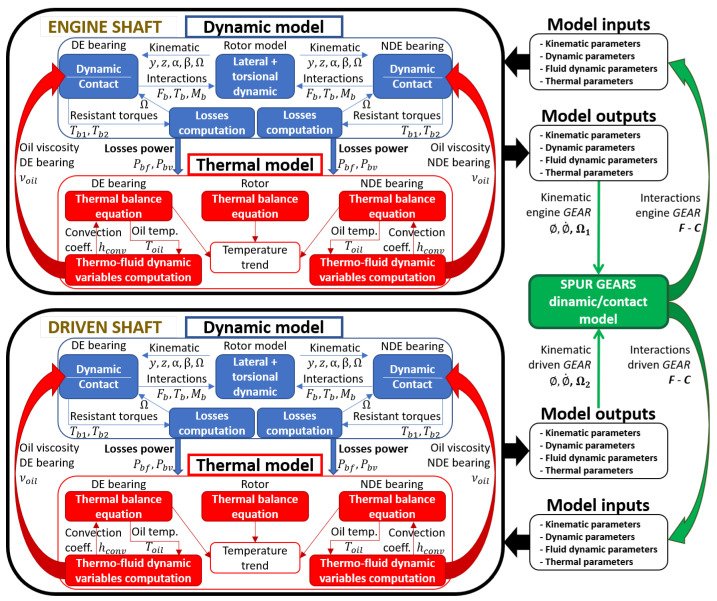
Schematic representation of the model architecture.

In this section, the structure of the developed numerical model representing the whole gearbox system is described. Figure 6 shows a synthetic representation of the part links of the model.

The gearbox system is composed of two rotors, linked between spur gears and supported by two axial-radial rolling bearings in the drive end (DE) position (bearing 1) and non-drive end (NDE) position (bearing 2), as described in the previous chapter. The whole model is able to predict the behavior of the complete gearbox system, both under transient and steady-state conditions, and consists of six main parts, where parts four and five represent the updates to the existing rolling bearing model:

1. Rotor dynamic model;
2. Rolling bearing dynamic and contact model;
3. Rolling bearing dissipation and power loss estimation;
4. Spur gear dynamic and contact model, characterized by a suitable combination of multibody and contact models, able to predict the dynamics of the elements and the related interaction forces and torques;
5. Spur gear dissipation and power loss estimation—through the proposed formulation, it is possible to estimate the different mechanical losses of the spur gears (mechanical torque losses due to friction and fluid dynamic effects) and calculate the related dissipated power;
6. System thermal model.

### 3.1. Rotor Model

**Figure 7:**
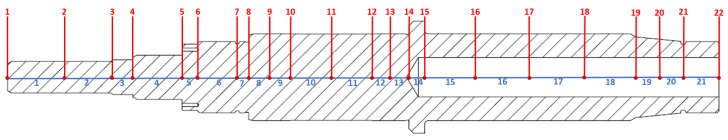
Discretization of the rotor in 3D BEAM elements.

**Figure 8:**
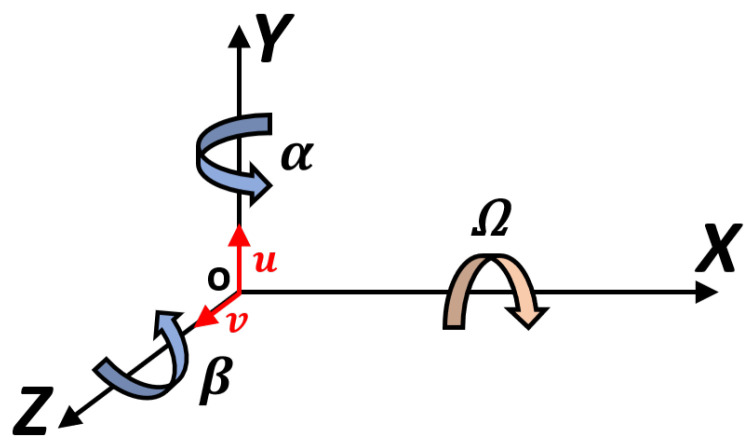
Rotor inertial reference system.

The rotor dynamics are described by the torsional and lateral dynamics. The torsional dynamics are modeled with a rigid model, while the lateral dynamics are represented by an FEM model (Figure 7). In the FEM model, each shaft element is considered a 3D BEAM (two nodes for each element); thus, the rotor geometry is represented in one dimension (Timoshenko’s beam), while the single rotor nodes are represented in two dimensions (plane motion—four DOF per node, see Figure 8) and the whole rotor is represented with 3D dynamic behavior [1].

For every shaft, the gears are modeled like added mass and inertia on the relative nodes on the shafts, with rigid links.

The model inputs are the bearing actions (forces and torques) $F_{b}$ and the external actions (forces and torques) $F_{e}$. This paper introduced also the actions in correspondence with the shaft gear node: $C_{\mathrm{gear}}$ and $F_{\mathrm{gear}}$. The kinematic outputs of the rotor model are the translational positions *y*, *z* and the angular rotations α, β for each rotor node.

The complete motion equation for the rotor lateral dynamics is as follows:

(1) $$M_{\mathrm{rot}}\ddot{x}+C_{\mathrm{rot}}\dot{x}+\Omega G_{\mathrm{rot}}\dot{x}+K_{\mathrm{rot}}x=F_{E}+F_{B}+F_{\mathrm{gear}},$$

where x contains the node variables, while $M_{\mathrm{rot}}$, $C_{\mathrm{rot}}$, $G_{\mathrm{rot}}$ and $K_{\mathrm{rot}}$ are, respectively, the mass, damping, gyroscopic and stiffness matrices of the rotor. The vector $F_{E}$ contains the external forces ($F_{e}$) acting on the rotor nodes, the vector $F_{B}$ contains the forces acting on the bearings $F_{b}$, while the vector $F_{\mathrm{gear}}$ contains the forces due to the gears.

**Figure 9:**
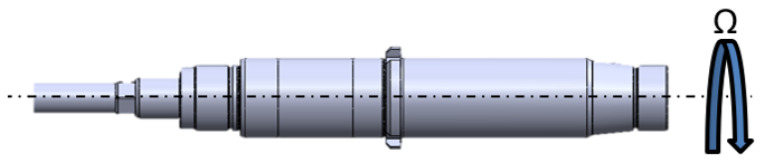
Rotational axis and degree of freedom for the torsional model.

Regarding the torsional dynamics, an approximate rigid torsional model was considered (Figure 9). This simplified model allows estimating the shaft rotational velocity Ω during the whole simulation.

The torques acting on the rotor are the torsional bearing moments (considering the seal friction moments), the air friction moment on the rotor and the gear moment (considering the gear friction moments), see Equation (2):

(2) $$I\dot{\Omega }=M_{b}+M_{wind}+M_{gear},$$

where *I* represents the torsional moment of inertia, $\dot{\Omega }$ is the rotor angular acceleration, $M_{b}$ is the torsional torques exerted by the two bearings on the rotor, which consist of the friction torque due to rotor-inner ring radial rub $T_{fr}$ and the friction torque due to rotor-inner ring axial rub $T_{rub}$, $M_{wind}$ represents the air friction moment on the rotor (neglected components moments) and $M_{gear}$ represent the moment the gears act on the rotor (considering the gear friction moments).

The two shafts are linked with the gear models, so the torsional model of the two shafts are also linked with each other. The angular velocity decrease due to friction: for this reason, in the model, it is possible set an input constant angular velocity for the engine shaft, while the driven shafts, with gears, accelerate with the engine shaft. The torsional dynamic equations of the gearbox system are the ones below:

(3) $$M_{m}-M_{f}=\frac{d(I\Omega )}{dt},$$

where $M_{m}$ is the engine torque, $M_{f}$ id the friction torque, *I* is the polar inertia moment of the rotational masses, with respect to the rotational axis, and $\dot{\Omega }$ is the angular speed rotation. Inertia *I* is composed of the following:

(4) $$I=I_{rot}+I_{gear},$$

where $I_{rot}$ is the rotor polar inertia moment and $I_{gear}$ is the gear polar inertia moment. In common application systems, the inertia is constant. For this reason, Equation (3) becomes:

(5) $$M_{m}-M_{f}=I\frac{d(\Omega )}{dt},$$

Moreover, below are the conditions needed to obtain angular velocity acceleration, deceleration or constant:

(6) $$M_{m}>M_{f}\Rightarrow \frac{d(\Omega )}{dt}>0,$$

(7) $$M_{m}<M_{f}\Rightarrow \frac{d(\Omega )}{dt}<0,$$

(8) $$M_{m}=M_{f}\Rightarrow \frac{d(\Omega )}{dt}=0,$$

**Figure 10:**
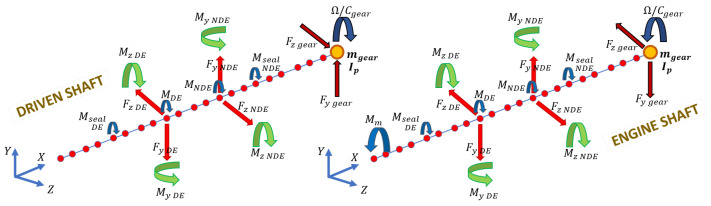
Force and torque agents on the shafts nodes.

In Figure 10, an image is presented which describes all force and torque agents on the shafts nodes, as listed above.

### 3.2. Rolling Bearing Mechanical Model

The rolling bearing model is used to predict the bearing dynamics and to evaluate the interaction actions between the rotor and the housing. Moreover, the estimation of the mechanical power losses is implemented in the bearing model to evaluate the temperature of its components.

The rolling bearing system and the relative numerical model were previously described by the authors in a preliminary paper [1]. Despite this, we will briefly describe the main parts of the bearing model.

**Figure 11:**
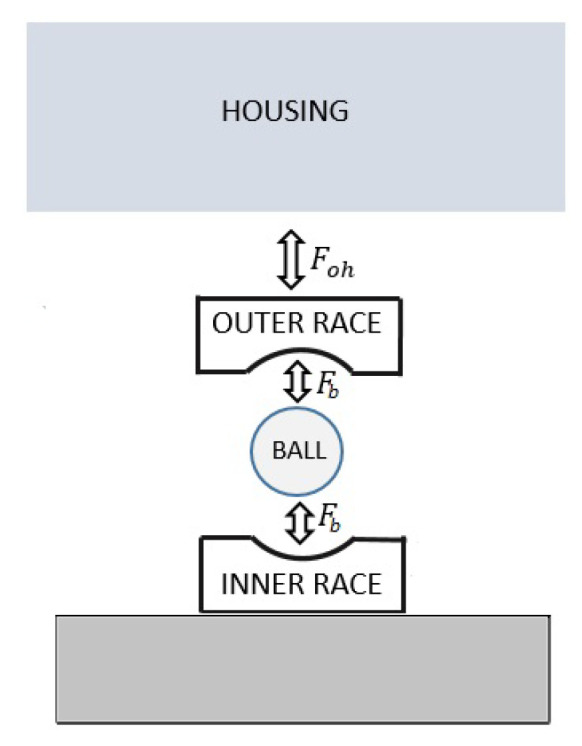
Schematic representation of the interactions between the elements of the bearing system.

With respect to the dynamical model, the outer ring dynamics are described with a planar motion (orthogonal to the shaft rotational axis) while the inner ring is supposed to be fixed to the rotor. In a rolling bearing, the link between the inner and outer ring dynamics is due to the ball elements, rolling in the races. In the bearing model used, the balls are modeled as force elements, so their dynamics have not been considered (Figure 11).

The estimation of the mechanical power losses in the rolling bearing takes into account the mechanical torque losses due to friction and fluid dynamic effects. The rolling friction evaluation is based on the Harris–Palmgren formulation [26], considering the dynamic forces acting on the bearing. The Harris–Palmgren formulation takes into account the bearing type and geometry, bearing load (static, dynamics and preload) and specific empirical coefficients.

The equations considered to model the mechanical power losses due to fluid dynamic effects are based on the Palmgren Theory [26,27]. This theory takes into account the oil lubricant viscosity, the rotor angular speed, dimensionless coefficients for the specific application and the type of lubrication.

In the end, the resultant dissipated torque acting on the bearings can be calculated as the sum of the single torque contributes due to outer ring contact, the rolling friction and the fluid dynamic effects.

### 3.3. Gear Mechanical Model

**Figure 12:**
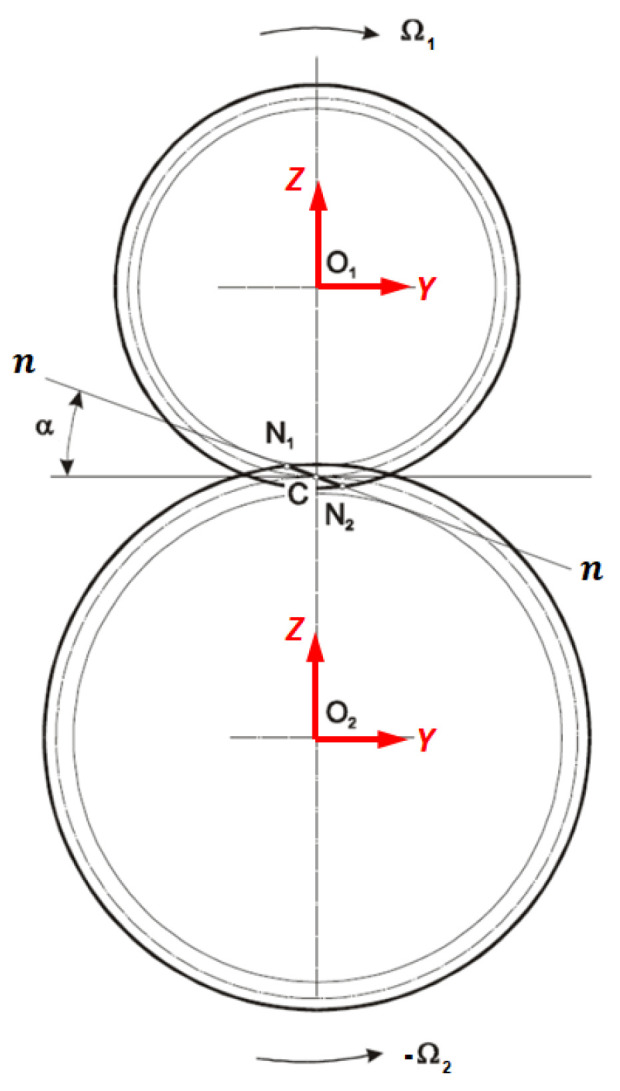
System references for gear teeth.

The gear mechanical model implemented in the gearbox model is the Ebrahimi gear model, from Ref. [5], as mentioned in the introduction, where the gear’s torsional degree of freedom was studied. The gears are considered similar to concentrated masses applied to the end of the rotors, in particular at the end node of the schematization elastic line rotor, while with the torsional dynamic rigid simulation, it is possible to obtain the torsional DOFs needed to calculate the engagement conditions between gears. The gears are placed in plane YZ (see Figure 8 and Figure 12); moreover, in the Ebrahimi gear model, the following is supposed:

1. Only one tooth is in contact during gear engagement;
2. The contact between teeth occurs along the contact line (or action line);
3. The teeth interpenetration δ.

**Figure 13:**
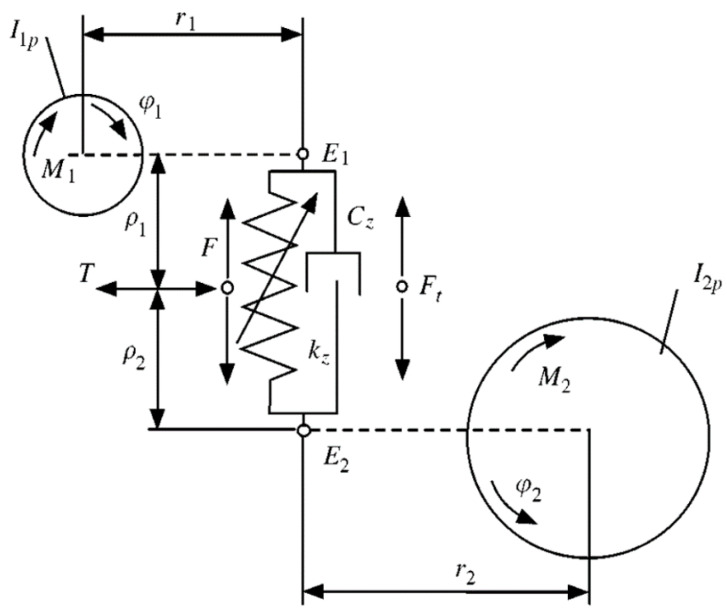
Modeling of teeth contacts with double parameters, stiffness *k* and damping *c*.

**Figure 14:**
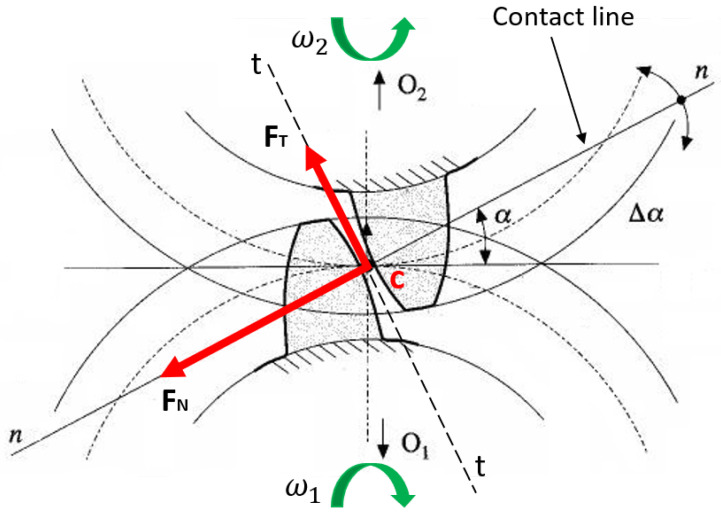
Teeth forces during contact with gears.

In the gear model, the relativity contact velocities between teeth are calculated as follows:

(9) $$v_{n}=\Delta \omega r_{b},$$

(10) $$v_{t_{i}}=\left(\right|\omega_{1}\left|+\right|\omega_{2}\left|\right)\alpha_{i}r_{p}\;i=1,2,\ldots ,k,$$

where i is contact teeth number—considering the previous hypothesis of the gear model, in this model, we consider only one couple of teeth to be in contact. In Equation (9), the normal velocity $v_{n}$, related to the contact point, is the same for every contact condition, so it is independent of the contact condition. Moreover, Δω is the different angular velocity between gears and $r_{b}$ is the base radius. In Equation (10), the tangential velocity $v_{t_{i}}$ is represented, related to every k contact situations, which takes into account the angular speed gears $\omega_{1}$ and $\omega_{2}$, the gear contact angle $\alpha_{i}$ and the primitive radius $r_{p}$. The tangential relative speed, with the constant friction coefficient defined, permitted the determination of the variable friction coefficient $\mu_{i}$ on the engagements teeth surfaces. Subsequently, the gear model calculates the interpenetration $\delta_{i}$ for every contact point. Thus, it is possible to calculate the normal contact forces $f_{n_{i}}$ using the stiffness and the damping in the contact point (Equation (11) and Figure 13):

(11) $$f_{n_{i}}=K_{i}\delta_{i}+C_{i}v_{n}\;i=1,2,\ldots ,k$$

where $K_{i}$ and $C_{i}$ are the respective stiffness and damping coefficient (see Figure 14). The interpenetration $\delta_{i}$ between teeth in contact is equal to zero in the case of pure rolling ($\delta_{i}=0$). However, because we also consider the teeth interpenetration to realize the motion transmission, the interpenetration $\delta_{i}$ can be explained with the following Equation:

(12) $$\delta_{i}=r_{p_{1}}\varphi_{1}+r_{p_{2}}\varphi_{2}$$

where $r_{p_{1}}$ and $r_{p_{2}}$ are the gears’ primitives radius, while $\varphi_{1}$ and $\varphi_{2}$ are the gears’ torsional DOF.

With the normal contact force $f_{n_{i}}$ and using the contact surface friction coefficient $\mu_{i}$, the tangential contact force $f_{t_{i}}$ is calculated as follows (see Figure 14):

(13) $$f_{t_{i}}=\mu_{i}f_{n_{i}}\;i=1,2,\ldots ,k$$

Thus, the contact force complete vector $F_{i}$ is calculated for every contact point with the projection of the normal and tangential contact forces, along direction *Y* and *Z* of the gears’ reference system (see Figure 12):

(14) $$F_{i}=\left[\begin{array}{c} F_{yi}\\ F_{zi} \end{array}\right]=\left[\begin{array}{c} (f_{n_{i}}cos\alpha_{i}-f_{t_{i}}sin\alpha_{i})cos\beta \\ f_{n_{i}}sin\alpha_{i}+f_{t_{i}}cos\alpha_{i} \end{array}\right]$$

where $\alpha_{i}$ is the gear’s angle pressure (same for the gears in contact) and β is the gear’s helix angle. In this paper, spur gears are studied, so the gear helix angle is equal to zero; thus, the expression used for $F_{i}$ is as follows:

(15) $$F_{i}=\left[\begin{array}{c} F_{yi}\\ F_{zi} \end{array}\right]=\left[\begin{array}{c} \left(f_{n_{i}}cos\alpha_{i}-f_{t_{i}}sin\alpha_{i}\right)\\ f_{n_{i}}sin\alpha_{i}+f_{t_{i}}cos\alpha_{i} \end{array}\right]$$

In this way, it is possible to calculate the torque $C_{i}$ corresponding to the contact condition. With the previous decomposition of force $F_{i}$ in the inertial system reference, the force $F_{zi}$ does not involve a torque; in contrary to $F_{yi}$, which generates a torque. The engine rotor is defined with subscript m, and the driven rotor is defined with subscript c; $\underline{n}$ and $\underline{t}$ are, respectively, normal and tangential gear unit vectors.

The normal force is directed along the gear engagement direction (action or contact line, see Figure 14), and it is a function of the engagement stiffness and damping parameter:

(16) $${\underline{F}}_{c\to m}^{n}=\left(k\cdot \delta +c\cdot \dot{\delta }\right)\cdot \underline{n}$$

(17) $${\underline{F}}_{m\to c}^{n}=-{\underline{F}}_{c\to m}^{n}$$

The normal force ${\underline{F}}_{c\to m}^{n}$ (Equation (16)) is the resistant force, acting on the engine gear, while the normal force ${\underline{F}}_{m\to c}^{n}$ (Equation (17)) is the motion force, acting on the driven gear. Moreover, k and c are, respectively, the contact stiffness and damping, δ is the teeth interpenetration and $\dot{\delta }$ is the temporal derivative. The verse of the normal force is driven by the interpenetration direction (see Figure 14).

The tangential force opposes the out direction with respect to the tooth, so the tangential force is opposite to $\underline{t}$:

(18) $${\underline{F}}_{c\to m}^{t}=-\left(\mu \cdot |{\underline{F}}_{c\to m}^{n}|\right)\cdot \underline{t}$$

(19) $${\underline{F}}_{m\to c}^{t}=-{\underline{F}}_{c\to m}^{t}$$

where μ is the constant dynamic friction coefficient; in this case, the two teeth slide on top of each other.

The tangential force ${\underline{F}}_{c\to m}^{t}$ (Equation (18)) is the resistant force, acting on the engine gear, while the tangential force ${\underline{F}}_{m\to c}^{t}$ (Equation (19)) is the motion force, acting on the driven gear. Obviously, the tangential force depends on the value of the friction coefficient μ; for this reason, the tangential force will be different with or without lubrication, due to the friction coefficient μ variation.

The calculation of the torque is reported in the following relative expression, where the vector product indicates the torques:

(20) $${\underline{M}}_{m\to c}^{O_{1}}=\left(C-O_{1}\right)\times {\underline{F}}_{m\to c}$$

(21) $${\underline{M}}_{c\to m}^{O_{2}}=\left(C-O_{2}\right)\times {\underline{F}}_{c\to m}$$

where ${\underline{M}}_{m\to c}^{O_{1}}$ is the torque with an $O_{1}$-like reduction center and ${\underline{M}}_{c\to m}^{O_{2}}$ is the torque with an $O_{2}$-like reduction center.

In all contact points, there is sliding between teeth, due to the gear relative movements, except for the intersection point between primitives diameters and action direction: the instantaneous rotation center is at that point. Adding to the previous indication, the friction coefficient is introduced to the model; for this reason, the forces are along the contact direction, as well as along the tangential direction, as explained above.

### 3.4. Stiffness, Damping and Interpenetration Condition

**Figure 15:**
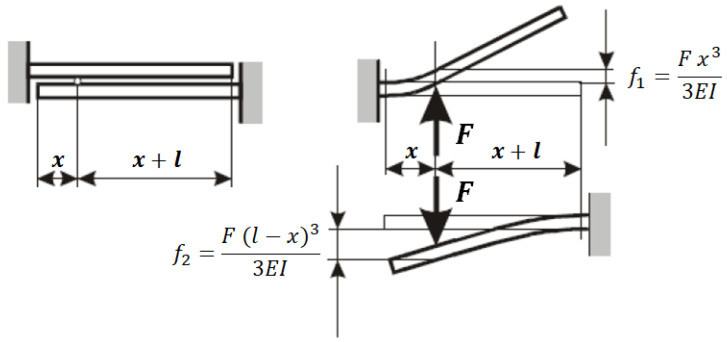
Variable teeth bending stiffness.

The stiffness engagement of a tooth couple is a function of the contact position. To understand this phenomenon, the teeth engaged are considered to be similar to beams (placed frontal each other) with a joint on one side of the beam, because the other tooth is on the other side. Moreover, the two beams (tooth gears) are loaded with the same force F and the terms “f1” and “f2” represent the displacements of the gear teeth in correspondence with the load direction with respect to the configurations that are not deformed (Figure 15):

To calculate the gear teeth contact stiffness, the teeth are considered to be similar to a rectangular section beam, with the following bending load:

(22) $$k=\frac{F}{x}=\frac{3EI}{L^{3}}$$

where *E* is the material elastic module (Young modules), *b* is the beam width, *h* is the beam thickness, *L* is the tooth length and *I* is the inertia momentum with respect to the neutral axis of the section (parallel to *b* dimension and orthogonal to *h* direction), obtained with the following equation:

(23) $$I=\frac{bh^{3}}{12}$$

Thus, with the previous expression for the rectangular section inertia momentum, it is possible to calculate the definitive expression for the bending stiffness:

(24) $$k=\frac{F}{x}=\frac{Ebh^{3}}{4L^{3}}$$

In Equations (22) and (24), the tooth stiffness is a function of the tooth length, i.e., the distance between the tooth loading position and the gear root diameter. These values, for both gears engaging together, change during the simulation and in particular during the meshing phase. Therefore, the “*L*” value in Equations (22) and (24) is not constant and the tooth stiffness changes during the meshing phase.

Instead, with respect to the damping, the state-of-the-art steel damping coefficient is assumed to be variable between 1% and 5% with respect to the stiffness engagement [3,4]. In the proposed model, a medium value equal to 3% of the stiffness engagement was considered. Therefore, the damping coefficient percentage is constant, while the damping coefficient value is variable due to the variable of stiffness engagement.

With respect to the interpenetration conditions (see Equation (12)), all the possible conditions are as follows:

- δ>0: teeth interpenetration, where the gears engage with motion transmission with a sliding motion between the teeth;
- δ=0: relative motion with pure rolling;
- δ<0: forces and torques equal to zero; otherwise, it would be similar to the driven shaft, which became the engine shaft.

**Figure 16:**
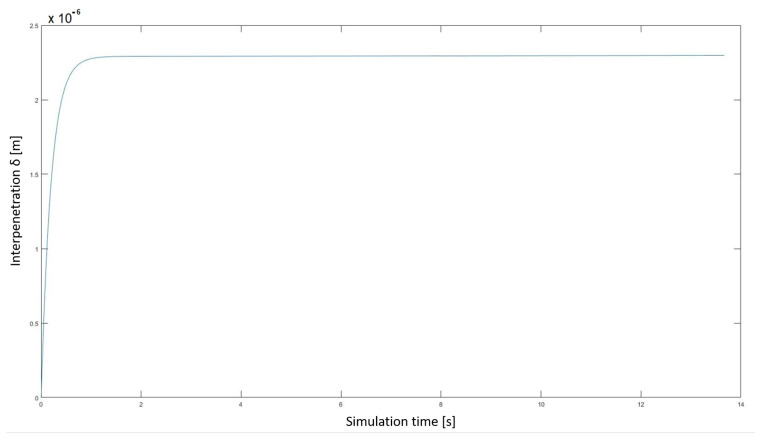
Interpenetration δ between tooth gears.

In Figure 16, the trend of interpenetration δ between tooth gears is represented.

After an initial transient, the interpenetration δ is stabilized to a value of $10^{-6}$ order. The component stiffness, damping and interpenetration are included in the normal forces.

### 3.5. Friction and Lubrication

Both friction and lubrication are aspects regarding the tangential contact force. With respect to friction, the model considers the Coulombian friction for dry surface states, where the friction coefficient does not depend on the load nor the contact area. The third claim has not yet been verified, i.e., that the friction coefficient does not depend on the sliding velocity. Furthermore, in the presented gear model, the friction coefficient has a constant value for contact steel/steel. The values presented in Table 2 are obviously only approximated, because the friction coefficient also depends on the surface roughness.

In the next-gear model development, it is possible to introduce the friction coefficient depending on the sliding velocity. In the first stage of the gear model, the friction coefficient does not depend on the sliding velocity because the friction dynamic coefficient is a tuning parameter for the model, the contact model is a non-linear contact model and the friction force is proportional to the normal force module and to the friction dynamic coefficient.

The lubrication is used to decrease the friction coefficient and the tangential force, other than the surface temperature. Obviously, the lubrication involves a resistant loss on the gears. Lubricated gears are not used the Coulombian friction laws, but are considered in other formulations.

### 3.6. Gears’ Fluid Dynamic Power Losses

The power losses present in a gearbox are of two types:

- Power losses dependent on the load;
- Power losses independent of the load.

The power losses dependent on the load are correlated with the motion torque and the engagement (sliding and rolling), other than the bearing losses. The power losses independent of the load are correlated with the fluid dynamic losses (windage losses, pumping losses and lubrication losses).

**Figure 17:**
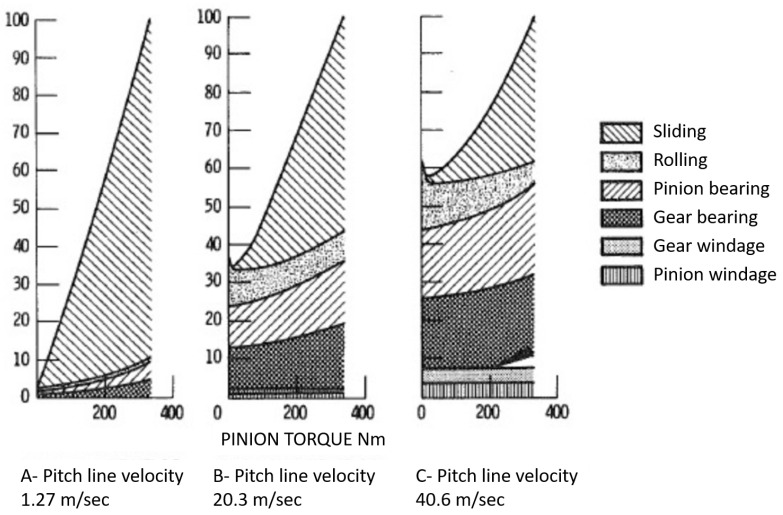
Ratio of power losses to full load loss (loss at peak of efficiency) at each speed %.

Usually, the power losses dependent on the load are higher with respect to the power losses independent of the load in the application with low speed and high torque transmission; instead, with higher angular speed, the fluid dynamic effect cannot be neglected, so in these cases, the power losses independent of the load are higher (see Figure 17).

With reference to the high-speed spur gear (aeronautical application), the most important fluid dynamic losses are:

- Windage losses;
- Pumping losses (Meshing losses);
- Oil injection losses.

### 3.7. Windage Losses

The windage losses are due to fluid dynamic losses on the gear surface. In particular, these are represented by aerodynamic forces (viscous and pressure) on the gear when it works in the air or in an air/oil cloud. In Equation (25) the windage-resistant torque is reported for a single gear [12,13]:

(25) $$M_{wind}=WPL=0.5\cdot c_{m\_wind}\cdot \rho_{air}\cdot \omega_{rot}^{2}\cdot r_{p}^{5}$$

where $\rho_{air}$ is the air density determined with perfect gases state equation, $\omega_{rot}\left(\frac{rad}{s}\right)$ is the angular speed rotation, $r_{p}$ is the gear primitive radius and $c_{m\_wind}$ is the windage coefficient that is determined experimentally with the function of the rotor angular velocity:

(26) $$c_{m\_wind}=1.0034\cdot \omega_{rot}^{-0.017}$$

where $\omega_{rot}$ is the angular speed rotation expressed in RPM. The formulation (26) originates from the Diab coefficient correlation [12], and depends on a lot of parameters, experimentally expressed in this coefficient. In the gear model, the windage torque is applied to both the gear torsional dynamics.

**Figure 18:**
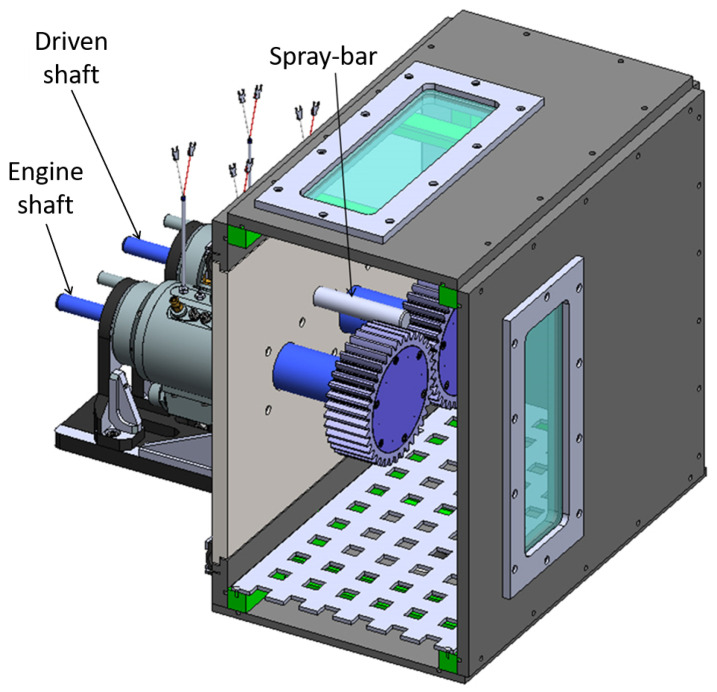
Depression test chamber to study the impact of windage losses.

In Equation (25), the test chamber is installed on the test rig. The test chamber includes the two gears engaged (Figure 18). The test chamber has a double purpose: to realize a depression volume with respect to the ambient pressure (to reduce the windage losses) and contain the lubrication oil. An appropriate formulation is used in the gear model for the experimental case (closed test chamber). The windage losses are dissipative losses and they are present every time the gear is not completely enclosed in a chamber volume. In the case study, since the gears are located in the under-pressurized test chamber, the shape of the casing strongly influences the dissipative component losses. The test chamber is intended to reduce these losses.

### 3.8. Meshing Losses

**Figure 19:**
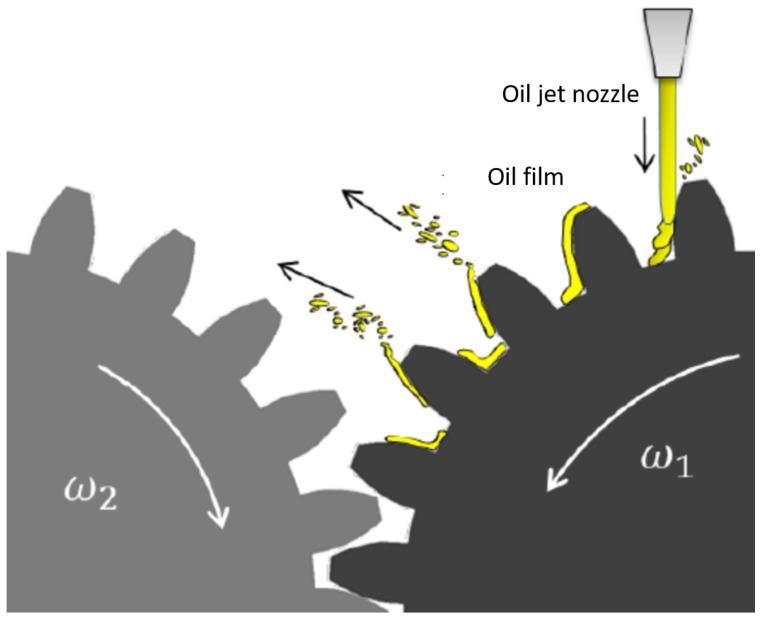
Oil jet nozzle, oil jet film and pumping losses due to oil present in the contact zone between teeth.

The meshing losses are related to the compression/expansion of the oil/air volume between teeth during the engagement, so the gears behave similar to a volumetric pump (Figure 19). This loss component is important with lubricated gears, but it is not negligible with gears moved in the air without lubrication. We can calculate the meshing resistant torque for a single gear using Equation (27) [12,13]:

(27) $$M_{mesh}=WPL=0.5\cdot c_{m\_mesh}\cdot \rho_{air}\cdot \omega_{rot}^{2}\cdot r_{p}^{5}$$

where $\rho_{air}$ is the air density determined with the perfect gases state equation, $\omega_{rot}\left(\frac{rad}{s}\right)$ is the angular speed rotation, $r_{p}$ is the gear primitive radius and $c_{m\_mesh}$ is the meshing coefficient that is determined experimentally in the function of the rotor angular velocity, calculated with the following equation:

(28) $$c_{m\_wind}=0.7179\cdot \omega_{rot}^{0.0387}$$

where $\omega_{rot}$ is the angular speed rotation expressed in RPM. The formulation (28) depends on a lot of parameters, experimentally expressed in this coefficient [12]. In the gear model, the meshing torque is applied to both gear torsional dynamics.

### 3.9. Oil Injection Losses

**Figure 20:**
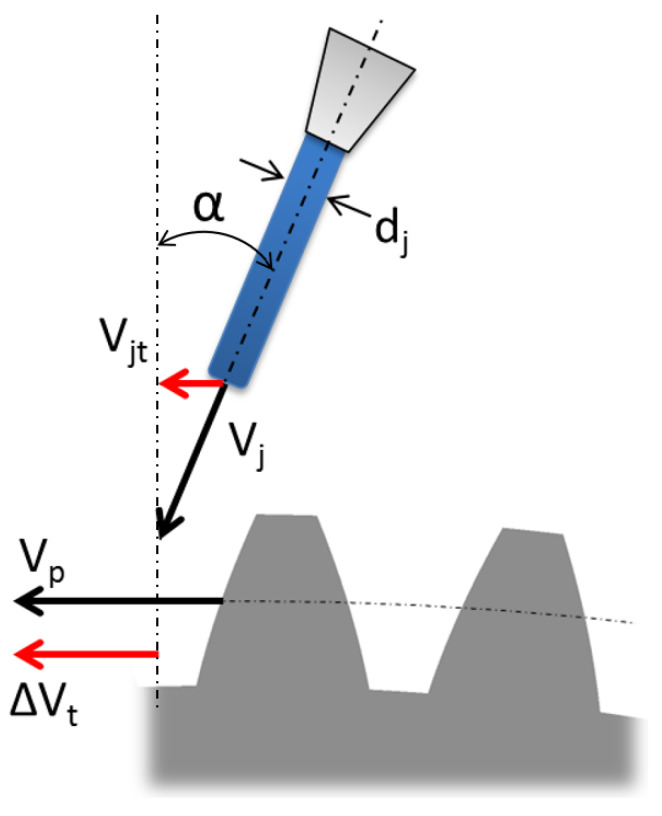
Oil jet lubrication modeling on gear.

The gears can be lubricated using different methods, depending on the pitch speed of the gear. For an aeronautical gearbox, where the pitch speed is very high (superior to $\left(10\frac{m}{s}\right)$, the better way to lubricate the gears is to use oil jet to lubricate the engagement zone and to refrigerate the teeth. The oil jet hits the tooth surface, removing the heat and creating an oil film on the surface to reduce friction. This oil film needs to be continuously powered because the centrifugal forces tend to expel the oil on the teeth. Thus, the oil injection losses are due to the transfer by the injection to the tooth gears. The torque losses due to the oil injection are calculated and validated with a 0D model [12,13] (see Figure 20).

The oil jet torque resistance is calculated with the time variation of the angular momentum during the interaction with the gear teeth:

(29) $$M_{qm}=\dot{m_{j}}r_{p}\left(\omega_{gear}r_{p}-V_{j}\sin\alpha \right)$$

where $\dot{m_{j}}$ is the oil mass flow injected on the gear teeth, $r_{p}$ is the gear primitive radius, $\omega_{rot}$ is the angular speed rotation, $V_{j}$ is the oil jet speed and α is the injection angle relative to the gear. The experimental test was realized with a radial oil jet $\left(\alpha =0\to \sin\alpha =0\right)$, and thus, Equation (29) became:

(30) $$C_{qm}=C\left(0\right)=\dot{m_{j}}\omega_{gear}r_{p}^{2}$$

The two most important oil injection modes are in-mesh and out-of-mesh. The first occurs when the gear oil jet is utilized before the engagement, and vice versa for the second type. The pros and cons of these methods have been described in the Introduction. In the experimental tests, the oil jet is utilized in the in-mesh mode. The oil injection torque resistance is precisely due to the mechanical action that the jet implies on the teeth. The most important hypothesis for Equation (29) is that the oil mass flow is accelerated for the same entity: this is not always true considering that the tooth surface is inclined and that the impact can occur on different gear radius and for different gear speeds. In addition, a part of the oil affects the upper part of the tooth, where it can be accelerated by viscous forces, not due to impact. Finally, an oil rupture occurs before the impact could alter the speed or reduce the mass flow.

### 3.10. Mechanical Power Losses Calculation

With the formulation of the different types of resistant torques, it is possible to evaluate the mechanical power loss due to the rolling bearing [1] (Equation (31)) and due to the gears (Equation (32)):

(31) $$\begin{array}{c} P_{bf}=M_{bf}\cdot \Omega \\ P_{bv}=M_{bv}\cdot \Omega \\ P_{b}=P_{bf}+P_{bv}. \end{array}$$

(32) $$\begin{array}{c} P_{contact}=M_{contact}\cdot \Omega \\ P_{wind}=M_{wind}\cdot \Omega \\ P_{mesh}=M_{mesh}\cdot \Omega \\ P_{qm}=M_{qm}\cdot \Omega \\ P_{gear}=P_{contact}+P_{wind}+P_{mesh}+P_{qm}. \end{array}$$

In the previous equation, $P_{contact}$ is the power loss due to normal and tangential contact forces between teeth, $P_{wind}$ is the power loss due to air resistance on the gear, $P_{mesh}$ is the power loss due to meshing loss for air/oil between teeth, $P_{qm}$ is the power loss due to the oil injection and $P_{gear}$ represents the gears’ total mechanical and fluid dynamic power loss.

### 3.11. Rolling Bearing Thermal Model

**Figure 21:**
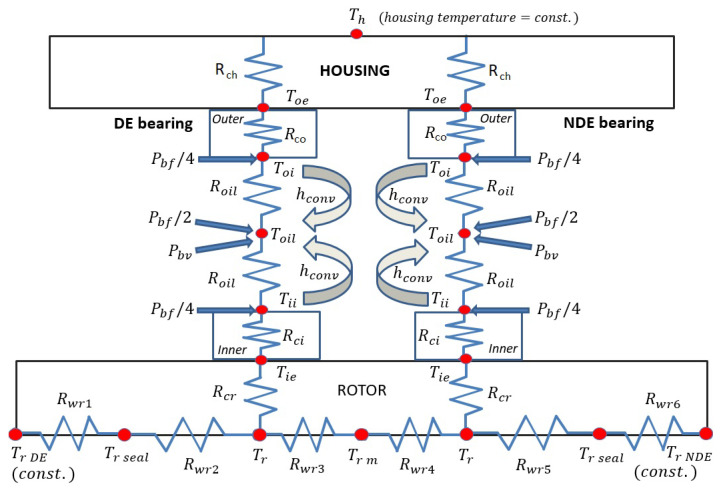
Schematic representation of the thermal network of the system.

As for the bearing mechanical model, the rolling bearing thermal model was already presented by the authors [1]; thus, we will only present the main concepts of the thermal model. The thermal model is composed of a thermal network with nodes connected through thermal resistances (Figure 21). Thus, a lumped parameter model was chosen to describe the effects of mechanical power losses on the rotor dynamic system, where the thermal resistances take into account both the geometrical shape of the elements, the materials and the corresponding value of conductivity.

Moreover, the mechanical power losses presented in the previous chapter, $P_{\mathrm{bf}}$ and $P_{\mathrm{bv}}$, are the inputs of the thermal model, and they determine the temperature values of each node (Figure 21).

Of course, the thermal balance equations for each node also take into account, in addition to the thermal resistances, the mass of the components and their specific heat. Moreover, for the oil nodes, the convection coefficient for the convection heat transfer and the variation of viscosity and density with the temperature are also considered.

The convection heat transfer and the calculation of the related $h_{conv}$ parameter are considered for the external node of the outer ring, the oil node and the internal node of the inner ring [28,29]. For the evaluation of the convection coefficient $h_{conv}$, the formulation of Gupta is considered [30]. In particular, for spherical elements in a fluid, convection heat transfer is generally expressed in terms of three dimensionless numbers (Nusselt Nu, Reynolds Re and Prandtl Pr numbers), with specific formulations, which were considered for the studied application.

### 3.12. Gear Thermal Model

In the gear thermal network, the heat exchange phenomena are as follows:

- Convective heat exchange with air external;
- Convective heat exchange with oil.

The gears have convective heat exchange with external air due to the windage effect and oil lubricant. The gear thermal network is considered one thermal node in the gear center line (one for every gear) and one thermal node in the contact zone, because at that point there are power losses due to teeth contact and the reduction of heat due to oil.

**Figure 22:**
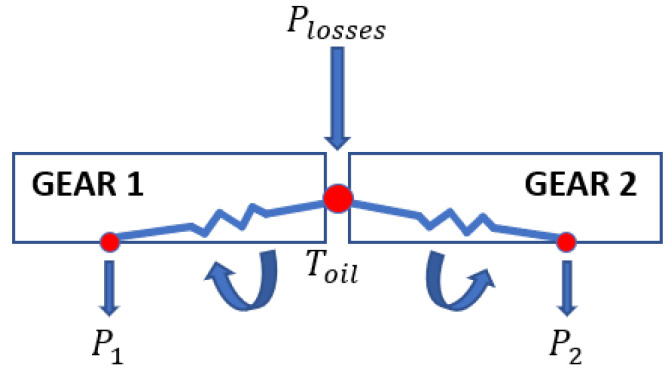
Gear thermal network.

The gear thermal network consists of the two previous nodes presented, and it is linked to the bearing network to disperse heat in the surrounding area. In Figure 22, the relationship between the thermal nodes and the power losses is presented. For every thermal network, nodes are calculated with the balance equation.

For the teeth thermal node, the power loss entering is due to the contact tangential force, the meshing losses and the oil injection. For the gear thermal nodes, the outgoing power loss is due to the windage losses.

### 3.13. Complete Gear–Rotor Model

**Figure 23:**
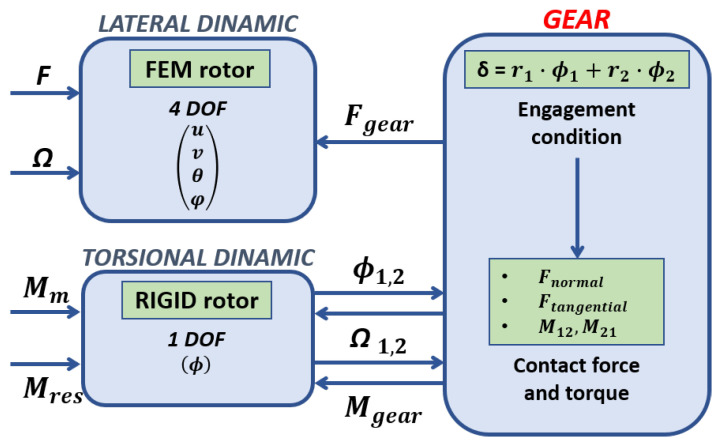
Complete gear model.

Figure 23 shows the complete gear dynamic for a single rotor, with the gear keyed on the end rotor. The gear model interacts with the lateral and torsional dynamics of both rotors due to the action and reaction law. The lateral dynamics are forced by the forces calculated from the gear model. The torsional dynamic is forced by all the torques deriving from the bearings and all the dissipative and non-dissipative effects of the gears. Obviously, the interaction is as if it were duplicated, since there are two coupled rotors; instead, the gear model is unique.

The gear model inputs are the physical and geometric parameters of the gearbox, in addition to those present in the rotor and the bearings, while the cinematic outputs are the angular displacements $\phi_{1}$$\phi_{2}$ and the relative angular speeds of the nodes where the gears are keyed on the rotors, as well as with the steady-state system and the rotors’ angular speeds.

**Figure 24:**
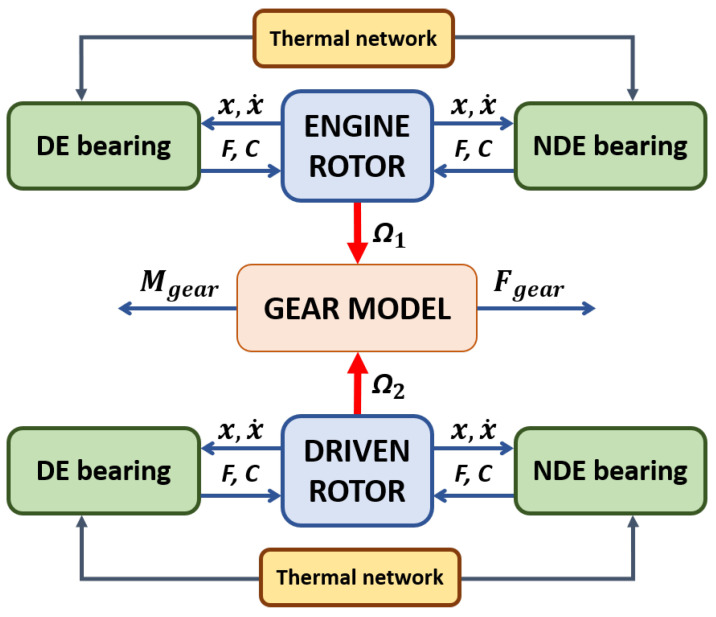
Complete gear–rotor model.

Figure 24 shows the complete model scheme (dynamic and thermal) with the rotor coupling with the gear model.

## 4. Experimental Tests

To obtain the matching between the simulation data and the experimental results, some experimental tests were planned on the test rig: obviously, the aim was to tune and validate the numerical model. To reach a deeper validation of the numerical model, the tests were characterized by different forms of flow rate gear lubrication (Table 3).

The tests were carried out in the presence of the coupling between the gears in the test chamber. Furthermore, the tests were carried out with lubricated gears and non-lubricated gears. In both cases, the experimental tests were performed with the same initial oil inlet temperature $T_{in}=50$ °C, the same lubricant oil flow rate bearing group ${\dot{m}}_{oil}=1.3$$\frac{L}{\min}$ (${\dot{m}}_{oil}=0.65$$\frac{L}{\min}$ for every single bearing on the engine and driven shafts) and the same rotor angular speed Ω, from 1000 RPM until 13,000 RPM (Table 3). Obviously, during the test, the engine angular speed was imposed, while the driven rotor speed had the same modules but opposite values (ratio equal to 1 between gears). Instead, the difference between the experimental tests is shown by the different ${\dot{m}}_{oil\_gear}$. The tests were performed with the following values of lubricant flow rate gears ${\dot{m}}_{oil\_gear}$: 0 $\frac{L}{\min}$, 1 $\frac{L}{\min}$, 3 $\frac{L}{\min}$, 5 $\frac{L}{\min}$ and 7 $\frac{L}{\min}$. Every previous value of the lubricant oil flow rate has to divide by four, the number of the holes on the spray bar, to obtain the oil mass flow for every spray bar hole. Finally, for tests with lubricated gears, the initial lubricating oil gear temperature was constant and equal to $T_{oil\_gear}=20$ °C.

In Table 4 the medium value of the test chamber temperature, test chamber pressure, the lubricant oil jet outlet temperature and the lubricant oil jet temperature are reported for every experimental test number. As can be seen, for every experimental test number, the test chamber pressure was constant at an ambient pressure of 101,509 Pa and the test chamber temperature oscillated between +/−3 °C.

Thus, the angular speed of the driving rotor, the inlet oil temperature and the oil flow rate for bearings and gears and for both rotors (driving and driven) are defined as input (Table 3). The physical quantities acquired for each test are the resistant torque measured on driving rotor $M_{b}$ and $M_{gear}$ due to the bearing group and gears dissipation effects, the mean oil outlet temperature $T_{oil}$, the mean average temperature of the bearing group of both rotors (driving and driven) $T_{oe}$ and the lateral acceleration $\ddot{y}$ on the DE bearing 1 and the NDE bearing 2 for both rotors. The temperatures of the external surfaces of the two bearings $T_{oe}$ are not reported individually, so it is experimentally observed that the difference between these two temperatures is negligible.

Every test starts from rotor speeds Ω equal from 13,000 RPM to 500 RPM with intervals of 1000 RPM for each test.

The experimental tests starting procedure takes into account the following steps: setting of the flow rates ${\dot{m}}_{oil}$ and ${\dot{m}}_{oil\_gear}$ and setting of the lubricant initial temperature $T_{in}$ and $T_{in\_gear}$; after that, the test bench chamber is closed and it is possible to start the electric engine. The rotor angular speed Ω increases slowly until 13,000 RPM (or lower values if necessary) and it is maintained constant until the thermo-couples registered constant values. In this way, the steady-state condition is achieved from a dynamic and thermal point of view and the output data can be acquired. After that, the rotor angular speed decreases until the next step and the data procedure acquisition is replicated. In Table 3, the values of the flow rates ${\dot{m}}_{oil}$ and ${\dot{m}}_{oil\_gear}$ and of the lubricant initial temperature $T_{in}$ and $T_{in\_gear}$ are listed for each test performed. The validation of some parameters of the model has been possible through the tests performed; in Table 5, some parameter values are reported.

## 5. Results and Discussion

This section presents the comparison between the tuning and the validation of the proposed model. The lubricant parameters of the model were already validated by [1], i.e., lubrication type coefficient $f_{0}$, thermal oil resistance $R_{oil}$ and lubricant viscosity $v_{oil}$. These parameters already had a good match with the data of the gears both with or without gear lubrication (Table 3).

### 5.1. Tuning of the Model Lubricant Parameters

In this subsection, the lubricant parameters of the model have been tuned. They are [26,27]:

- $f_{0}$: lubrication type coefficient;
- $R_{oil}$: thermal oil resistance;
- $v_{oil}$: lubricant viscosity value.

**Figure 25:**
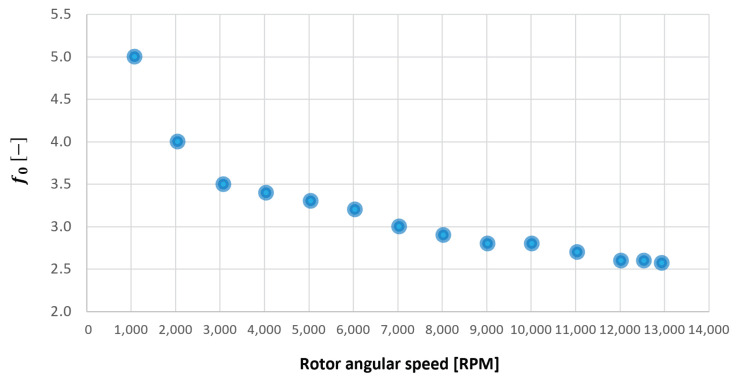
Lubricant type coefficient $f_{0}$ trend as a function of the rotor speed Ω, for a gearbox with gears mounted on shafts.

**Figure 26:**
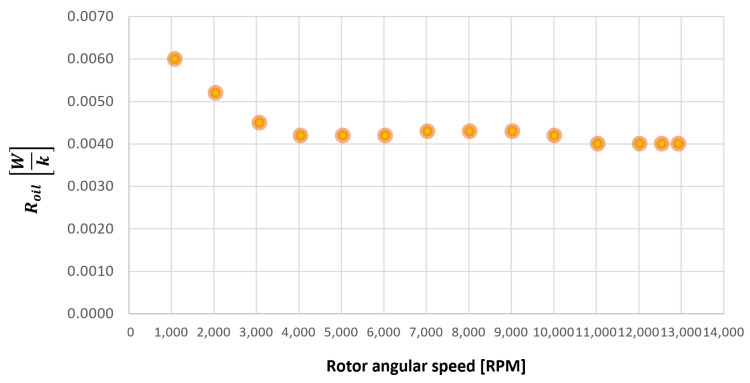
Lubricant thermal resistance $R_{oil}$ trend as a function of the rotor speed Ω, for a gearbox with gears mounted on shafts.

**Figure 27:**
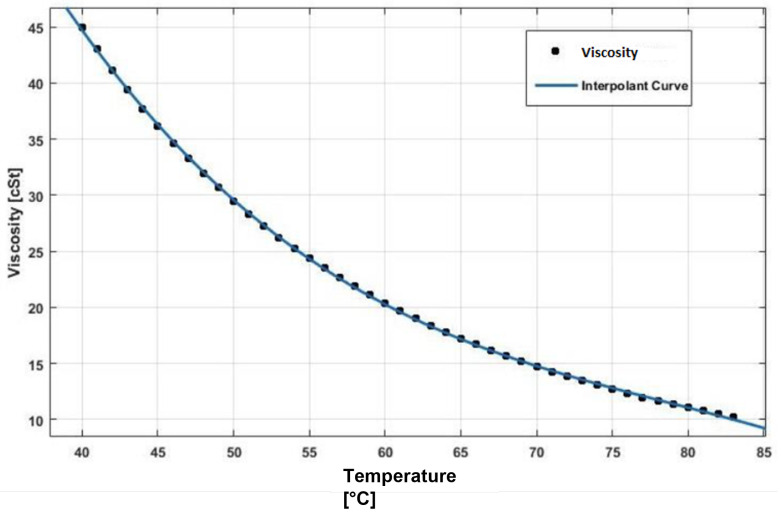
Lubricant viscosity $v_{oil}$ trend as a function of the oil temperature $T_{oil}$.

All the previous parameters were already introduced by Giannetti [1]; in fact, to start the tuning process of the model lubricant parameters for the gearbox with gears mounted on the shafts, the authors started by already-obtained tuning parameters. To find the correct, necessary tuning parameters for the gear model, only a few modifications to the previous tuning parameters were found to be needed by Giannetti [1]. The tuned values of these parameters have been obtained through experimental test 1, minimizing the errors between simulated and experimental outputs, which are the resistant torque $M_{b}$ + $M_{gear}$, the mean oil outlet temperature $T_{oil}$ and the mean bearing temperatures $T_{oe}$. The functions obtained for $f_{0}$, $R_{oil}$ and $v_{oil}$ are plotted in Figure 25, Figure 26 and Figure 27 as a function, respectively, of the oil temperature $T_{oil}$ and the rotor speed Ω.

**Figure 28:**
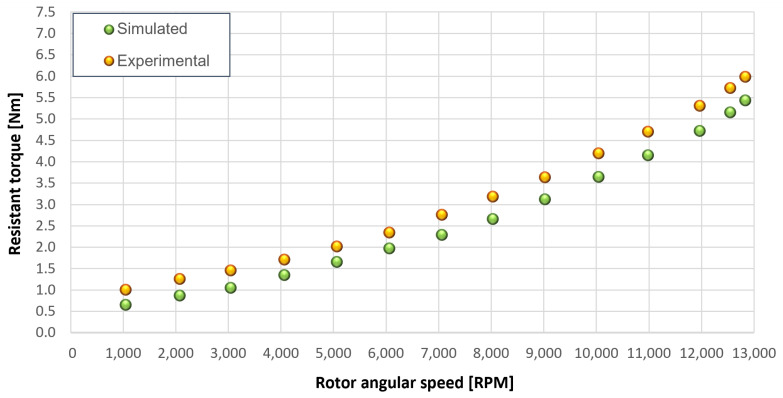
Comparison between experimental and simulated resistant torque $M_{b}$ + $M_{gear}$ for tests 1 without gear lubrication, see Table 3.

**Figure 29:**
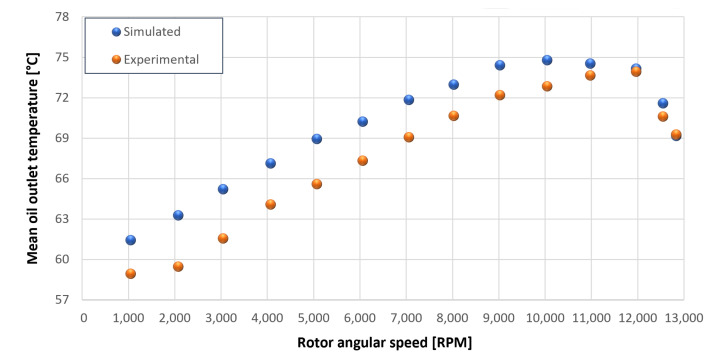
Comparison between experimental and simulated mean oil outlet temperature $T_{oil}$ for tests 1 without gear lubrication for engine rotor, see Table 3.

**Figure 30:**
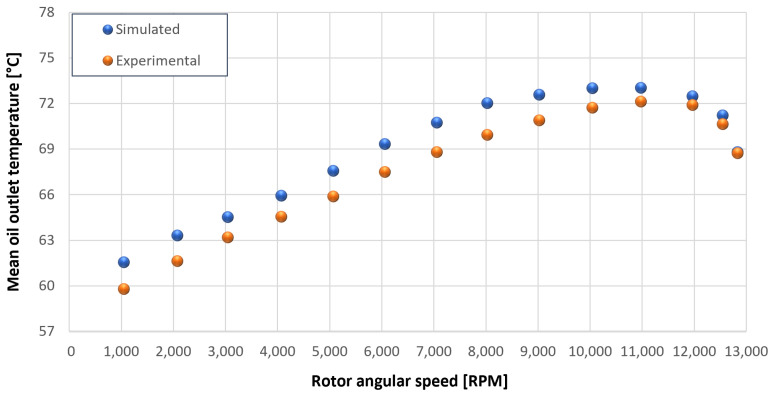
Comparison between experimental and simulated mean oil outlet temperature $T_{oil}$ for tests 1 without gear lubrication for driven rotor, see Table 3.

**Figure 31:**
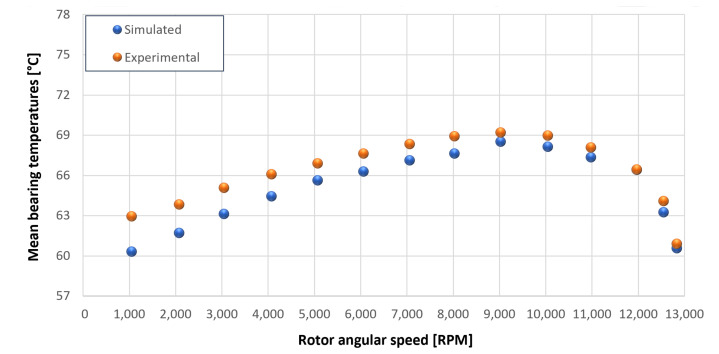
Comparison between experimental and simulated mean bearing temperatures $T_{oe}$ for test 1 without gear lubrication for engine rotor, see Table 3.

**Figure 32:**
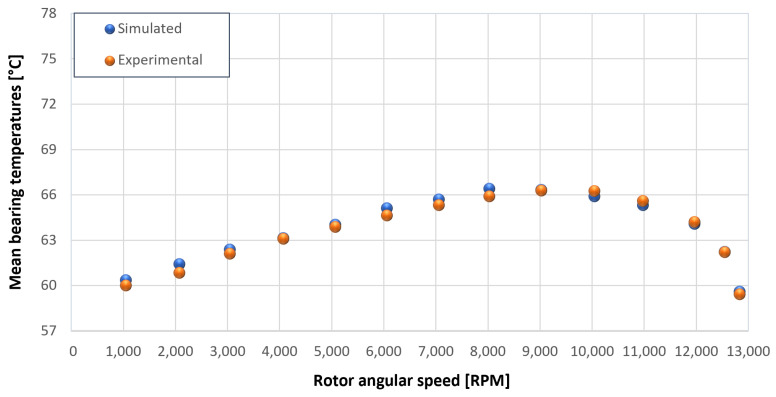
Comparison between experimental and simulated mean bearing temperatures $T_{oe}$ for test 1 without gear lubrication for the driven rotor, see Table 3.

The trends for the values of $f_{0}$, $R_{oil}$ and $v_{oil}$ have been already detailed by Giannetti [1]; the basic assumptions and the system behavior interpretation are still valid also with the introduction of the gears. Moreover, the behavior of the system with the gears (in terms of resistant torque $M_{b}$ + $M_{gear}$, mean oil outlet temperature $T_{oil}$ and mean bearing temperatures $T_{oe}$ on the DE bearing 1 and the NDE bearing 2) obtained with the optimization of the previous parameters are described in Figure 28, Figure 29, Figure 30, Figure 31 and Figure 32 as a function of the rotor speed Ω.

### 5.2. Model Validation: Comparison between Experimental Data and Simulated Results

In this subsection, the comparison between experimental data and simulated results is shown. The results are presented in different operating conditions and in particular with different gear lubricant flow rates ${\dot{m}}_{oil\_gear}$ (Table 3). As in the previous case without gear lubrication, the experimental data for each test are compared with the simulations provided by the proposed model in terms of the resistant torque $M_{b}$ + $M_{gear}$, mean oil outlet temperature $T_{oil}$ and mean bearing temperatures $T_{oe}$ on the DE bearing 1 and the NDE bearing 2 (see Figure 33, Figure 34, Figure 35, Figure 36, Figure 37, Figure 38, Figure 39, Figure 40, Figure 41, Figure 42, Figure 43, Figure 44, Figure 45, Figure 46, Figure 47, Figure 48, Figure 49, Figure 50, Figure 51 and Figure 52).

The proposed model reproduces well the mechanical power losses and the related thermal effects of the rolling bearing-rotors-gears system. Despite this, a few differences between experimental data and simulated results are still present. For instance, some overestimation of the bearing temperature $T_{oe}$ can be observed for low rotor speed values: this can be explained by the test rig chamber heating at the end of the tests and so with a lower air heat exchange.

Between tests with and without gear lubrication, there is no substantial difference in the resistant torque. The unique difference is that in Test 1 (“dry” wheels, without gear lubrication) and in Test 2 (gear lubricant mass flow ${\dot{m}}_{oil\_gear}=1$$\frac{L}{\min}$), there is a slight increase in the resistant torque in the lubricated case study. This makes it clear that the oil action is a factor in lowering the temperature of the system, and therefore, dissipates the generated heat, but from a mechanical point of view, it creates a small increase in resistance during meshing. The bearing temperatures decrease with the increase of the lubricant mass flow of the gears; however, it has been observed that for the lubricated case study, the bearing temperatures are higher with respect to the “dry” case study. Only with a gear lubricant mass flow ${\dot{m}}_{oil\_gear}$ higher than 5 $\frac{L}{\min}$ are the bearings temperatures comparable to the first case (“dry” Test 1). Regarding the oil inlet temperatures, the same values are obtained both with and without lubrication (see Table 3). The oil outlet temperatures from the bearings units (mounted on the engine and driven shafts) increase as the lubrication mass flow of the gears’ increase: the bearings have to dissipate a high heat quantity with respect to the “dry” gears and they are more stressed. Furthermore, it can be noted that the bearing temperatures on the driving rotor are slightly higher with respect to the driven one; there is also an asymmetry of the temperatures between the DE and NDE side, with some degree of difference. Finally, with a higher gear lubricant flow rate, the same gears disperse more heat and the bearings work at lower temperatures. Thus, the bearings dissipate very well in terms of temperature in both test modes, which suggests that they are the true dissipative contribution of the entire system. The gears, if designed in appropriate ways, have very little dissipative contribution: the model consolidates this hypothesis.

Indeed, the standard concept of good design for the toothed wheel means high DIN quality and low teeth roughness. Looking forward, with lower power losses due to the gears, the whole rotor-dynamic system has important benefits, such as lower bearing temperature.

These slight differences are due to the simplified hypotheses considered in the proposed model.

The rolling bearings are modeled through a lumped parameter model for a satisfactory compromise between accuracy and numerical efficiency. The same approach is used to describe the gears’ behavior and their coupling. Thus, many three-dimensional (3D) lubrication phenomena related to the gear meshing and to the bearing elements’ rollings on their rings are not fully considered.

On the other hand, the model considers only the flexibility of the rotor and neglects the others, such as the effect of the flexibility of supporting structures and basements: this might be another possible reason for discrepancies. Moreover, some heat exchange component has been neglected (i.e., convection between shafts and air) and the bearing temperature is, consequently, overestimated, especially at high speeds.

Finally, the electric motor dynamics have also not been considered in the model, and they could contribute to the differences between experimental and simulated results.

### 5.3. Resistant Torque Components

In the previous graphs, it is evident that good results are obtained for the resistant torque and the oil lubricant temperature leaving the bearing sets. Regarding the bearing temperature, however, there are slight overestimations with respect to the experimental values both with “dry” gears and lubricated gears, in particular at the low angular speeds of the spindles, with the same lubricant mass flow on the bearing side. This small overestimation of the bearing temperatures can be hypothetically explained by the simplification adopted by neglecting some heat exchange components which are present in the mechanical system and which leads to a drop in the bearings’ temperature at low spindle rotation speeds. These heat exchange components are not negligible if the powers involved in the mechanical system are comparable to the neglected one: this occurs when the dissipated powers that generate heat in the system are small; an eventuality that occurs if the injected oil is at very high temperature and the spindle speed is low. It can, therefore, be concluded that the obtained results by the model are very useful and significant, as they allow us to know the resistant torque components due to the bearing units and the gears (friction, lubrication, windage, meshing, oil jet); thus, they are quite accurate.

**Figure 53:**
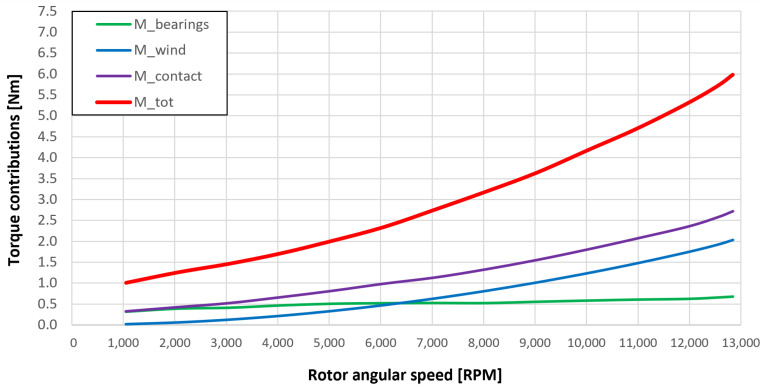
Comparison between torque contributions of test 1 (without gear lubrication, see Table 3).

**Figure 54:**
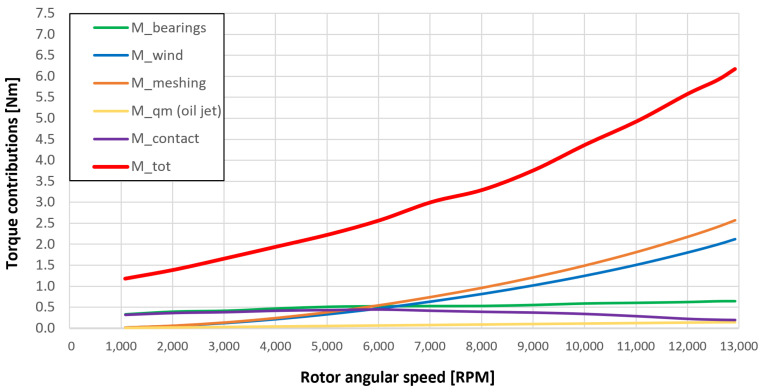
Comparison between torque contributions of test 2 (with a gear lubrication flow rate equal to 1 $\frac{L}{\min}$, see Table 3).

**Figure 55:**
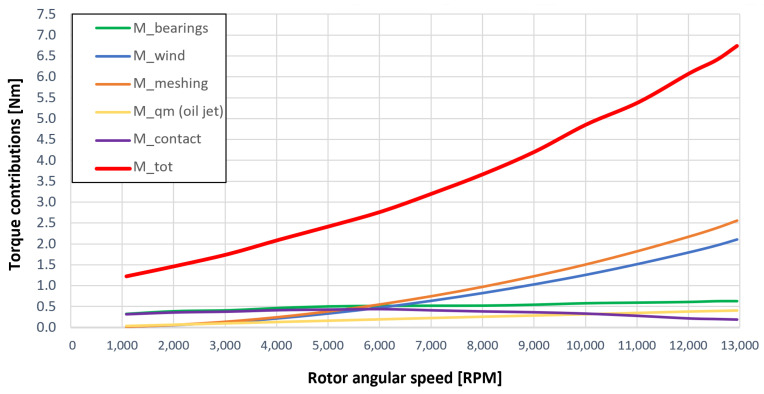
Comparison between torque contributions for test 3 (with a gear lubrication flow rate equal to 3 $\frac{L}{\min}$, see Table 3).

**Figure 56:**
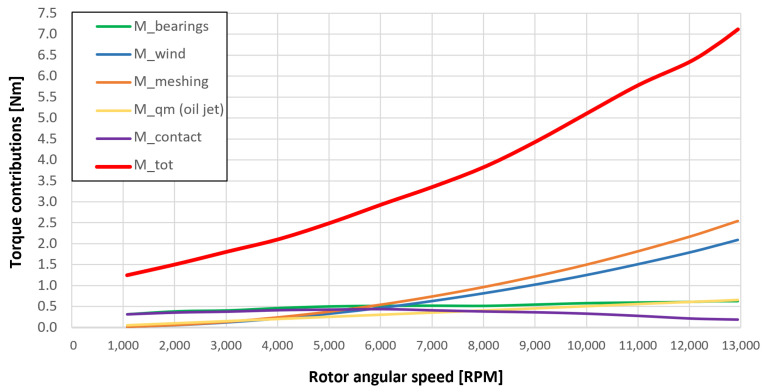
Comparison between torque contributions for test 4 (with a gear lubrication flow rate equal to 5 $\frac{L}{\min}$, see Table 3).

**Figure 57:**
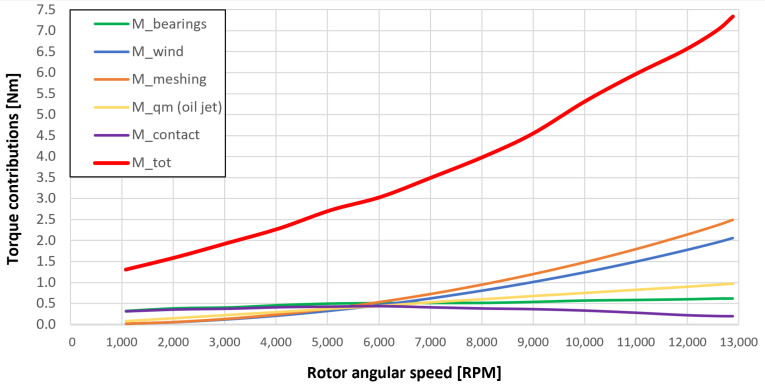
Comparison between torque contributions for test 5 (with a gear lubrication flow rate equal to 7 $\frac{L}{\min}$, see Table 3).

Figure 53, Figure 54, Figure 55, Figure 56 and Figure 57 show the torques’ contributions as a function of the angular speed rotation and for the various operating conditions. For the “dry” gears, the normal force contributions (stiffness and damping) and tangential friction were considered, while for lubricated gears, the model remained unchanged with only the modification of the tangential force components, no longer regulated by the friction modeling but with the presence of the lubricant. It can be seen that windage losses have a value approximately comparable to the meshing losses. It can also be seen that the mechanical torques gears due to the “dry” gears are quite significant, while in the lubricated case, these torques can be neglected compared to the friction case, with prevalent meshing and windage losses. Therefore, as could have been hypothesized in the preliminary phase for lubricated gears, the friction contribution is negligible compared to that due to fluid-dynamic losses.

The obtained results guarantee the model validity for the general behavior representation of the gearbox. The study allowed the estimation of the resistant torque of the considered system, with the aim of intervening in order to reduce the various kinds of losses. The following list describes the complete legend of the plotted resistant moment in the previous graphs:

- $M_{b}$: bearings torque;
- $M_{wind}$: windage torque;
- $M_{mesh}$: meshing torque;
- $M_{qm}$: oil jet moment torque;
- $M_{contact}$: torque due to the force (friction/lubrication) of the gears;
- $M_{gear}=M_{wind}+M_{mesh}+M_{qm}+M_{contact}$: total resistance torque due to the gear;
- $M_{tot}=M_{b}+M_{gear}$: experimental moment resistant, understood as the sum of all the previous contributions.

### 5.4. Computational Efficiency

After having spoken about the numerical efficiency of the proposed model, Table 6 explains its computer performances.

The compromise between accuracy and computational burden led the bearing-gear model to be used in a complex multibody system, involving many mechanical components.

## 6. Conclusions

In this work, the aim was to explore the mechanical and thermal behavior of a complex system, such as the one composed of rolling bearings and gears. In this respect, the estimation of the main mechanical power losses and the related thermal effects has been carried out and the proposed model has been validated through experimental tests completed on a specific test rig. The good agreement of the comparison between simulated results and experimental outputs confirms the goodness of the starting hypotheses and assumptions.

The current research paper focused on the aeronautical field, specifically the study of an integrated mechanical system with rolling bearings and toothed wheels, to tune a numerical efficient model with the experimental data, taking into account the main physics that influence the mechanical behavior, such as the dynamic and thermal aspects. Therefore, the aim of the model is to reach a good compromise between numerical efficiency, in terms of time-consuming reduction, and model simplification, maintaining the capability to represent the complex physics of important mechanical components, such as bearings, gears and rotor.

In particular, the model is capable to reproduce the resistant torque trend acting on the engine rotor of a system consisting of two rotors coupled by gears. In addition, it also reproduces each temperature of the bearing units that support both rotors. The model validation was carried out by comparing the experimental data with those measured in the simulation. Using the previous validation carried out for a single rotor [1], the coupling by means of the two gears has now made it possible to intervene with not only the characteristic parameters of the bearings, but also on gear parameters with respect to both the stiffness and the damping of meshing or the friction coefficient.

In the end, a balance between accuracy and numerical efficiency [25] has been pursued: this is important to predict simulations of real and complex systems operating for a long time period. Future research will focus on including other physical aspects in the proposed model that have been neglected in the first version of the model. In particular, it is possible to enhance the rolling bearing model with 3D effects, the flexibility of the main support, the electric motor’s effects, the introduction of the rotor axial dynamics and the wear modeling in the contact points. Moreover, environmental effects, such as noise, vibration and shocks, might also be considered in the updated model in addition to the wear, because all these aspects might affect the performance of the rotor system. In this context, obviously, the introduction of external torques and loads will also be considered to complete the model of the whole system and in particular the power losses and the power losses’ load dependency.

With respect to the gear modeling, the possible future developments concern both dynamic and thermal behavior. Regarding the dynamic model of the gears, both constant gear stiffness/damping and constant friction coefficient were considered: in the future, a periodic variable stiffness may be inserted. The same is true for the friction coefficient, which can be considered variable and dependent on the sliding speed (Hertzian friction) between the relative speeds in contact point. The developed model could be expanded by also implementing wear models that allow estimating the gears’ life.

These models might improve future developments of the proposed model because it will be possible to define the gear life and how the contact forces change during the gear life, thanks to knowledge of the worn teeth geometry. Moreover, in this way, the estimation of the power losses will also be more precise. In this ambit, the study of different gear widths and modules can also be investigated, with the aim to characterize different levels of heat exchange due to the gears and the consequence on the bearing temperatures of the system.

In addition, the dynamic and thermal model of the two gears can be used with the introduction of an applied load, to study the system behavior only with model simulations and not with experimental tests.

The improvements the authors can introduce in the model to enhance both the dynamic and thermal behavior of gear modeling are the introduction of a 3D formulation for the bearings and model the axial dynamics of the rotor and of the gears; with respect to the thermal behavior, different formulations can be introduced about the thermal exchange between gears and oil, taking into account when, in specific operation conditions, the presence of oil foam can become non-negligible.

## Figures and Tables

**Figure 33:**
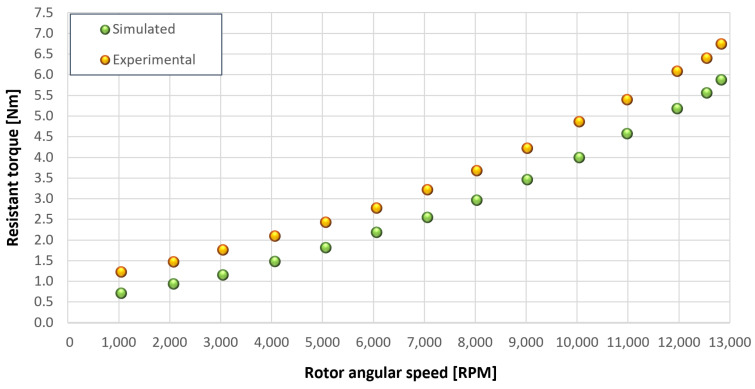
Comparison between experimental and simulated resistant torque $M_{b}$ + $M_{gear}$ for test 2 with a gear lubrication flow rate equal to 1 $\frac{L}{\min}$, see Table 3.

**Figure 34:**
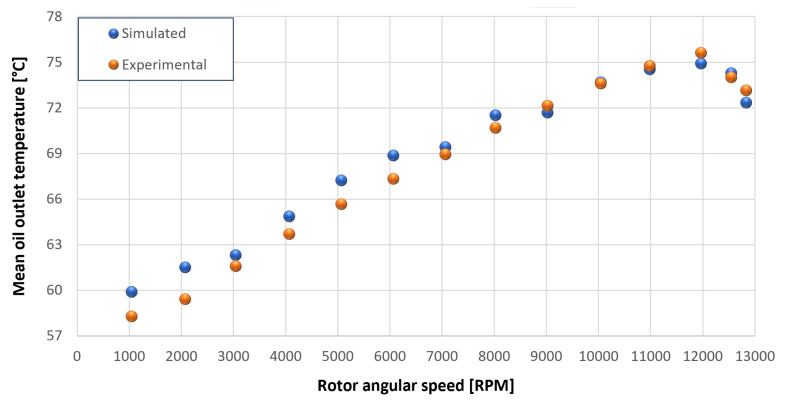
Comparison between experimental and simulated mean oil outlet temperature $T_{oil}$ for test 2 with a gear lubrication flow rate equal to 1 $\frac{L}{\min}$ for engine rotor, see Table 3.

**Figure 35:**
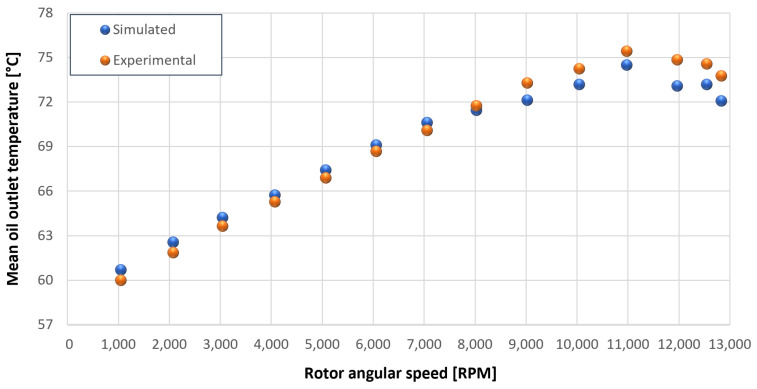
Comparison between experimental and simulated mean oil outlet temperature $T_{oil}$ for test 2 with a gear lubrication flow rate equal to 1 $\frac{L}{\min}$ for driven rotor, see Table 3.

**Figure 36:**
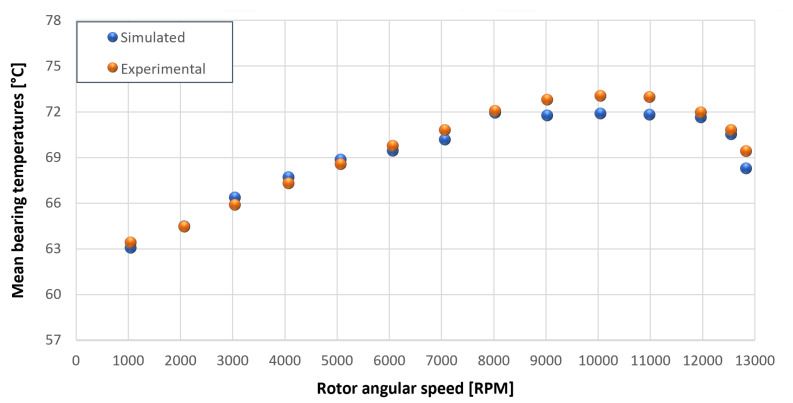
Comparison between experimental and simulated mean bearing temperatures $T_{oe}$ for test 2 with a gear lubrication flow rate equal to 1 $\frac{L}{\min}$ for engine rotor, see Table 3.

**Figure 37:**
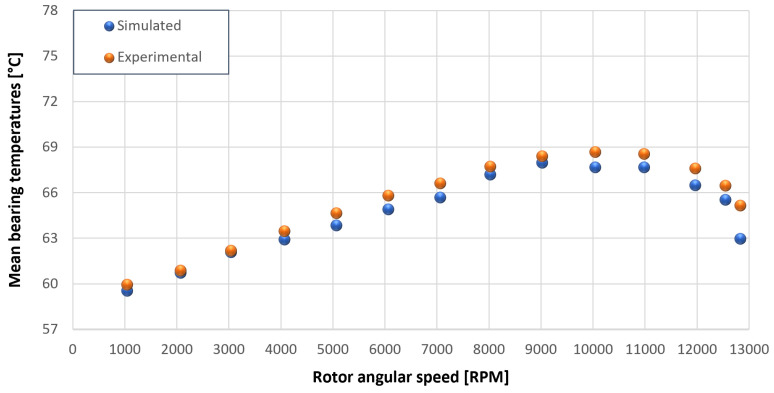
Comparison between experimental and simulated mean bearing temperatures $T_{oe}$ for test 2 with a gear lubrication flow rate equal to 1 $\frac{L}{\min}$ for driven rotor, see Table 3.

**Figure 38:**
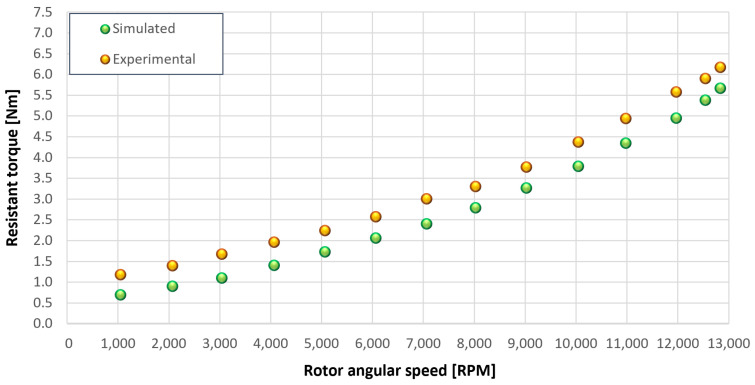
Comparison between experimental and simulated resistant torque $M_{b}$ + $M_{gear}$ for test 3 with a gear lubrication flow rate equal to 3 $\frac{L}{\min}$, see Table 3.

**Figure 39:**
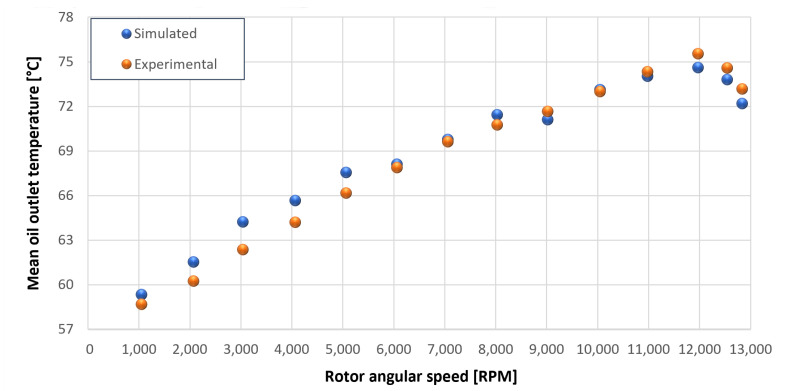
Comparison between experimental and simulated mean oil outlet temperature $T_{oil}$ for test 3 with a gear lubrication flow rate equal to 3 $\frac{L}{\min}$ for engine rotor, see Table 3.

**Figure 40:**
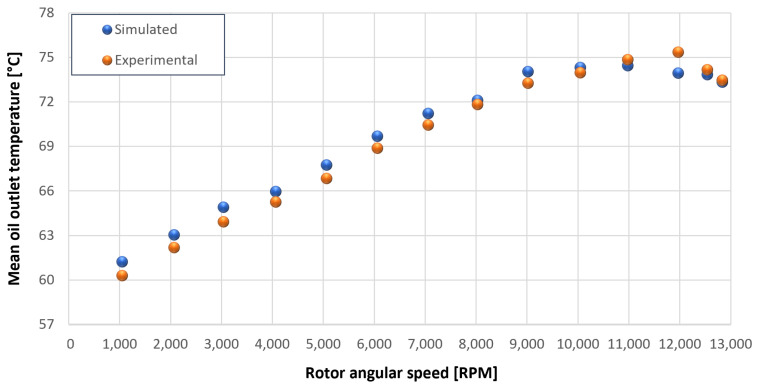
Comparison between experimental and simulated mean oil outlet temperature $T_{oil}$ for test 3 with a gear lubrication flow rate equal to 3 $\frac{L}{\min}$ for driven rotor, see Table 3.

**Figure 41:**
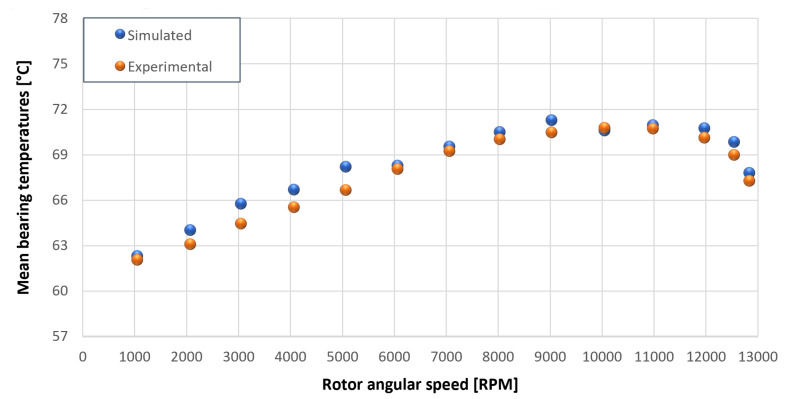
Comparison between experimental and simulated mean bearing temperatures $T_{oe}$ for test 3 with a gear lubrication flow rate equal to 3 $\frac{L}{\min}$ for engine rotor, see Table 3.

**Figure 42:**
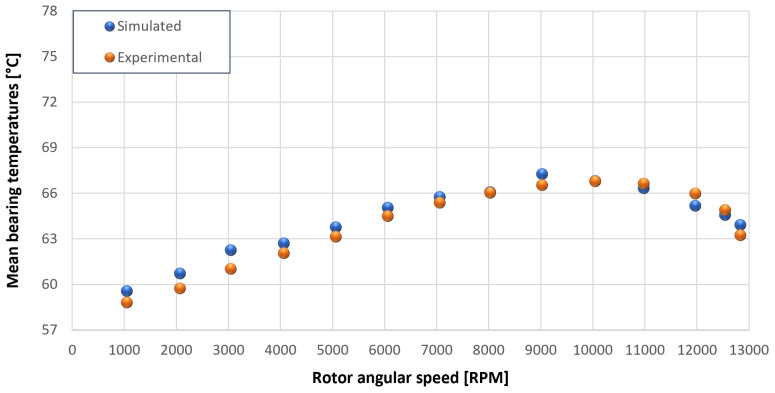
Comparison between experimental and simulated mean bearing temperatures $T_{oe}$ for test 3 with a gear lubrication flow rate equal to 3 $\frac{L}{\min}$ for driven rotor, see Table 3.

**Figure 43:**
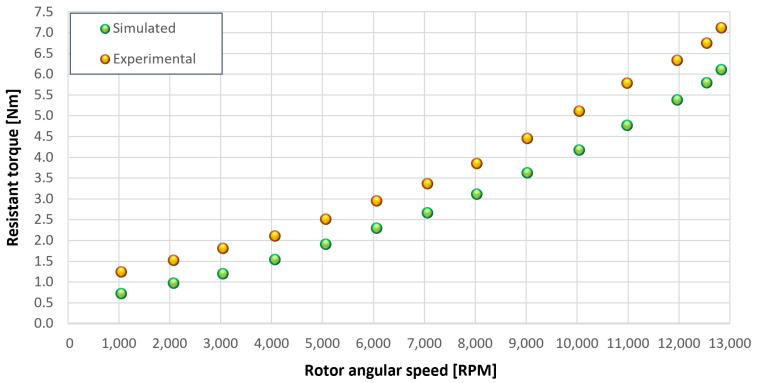
Comparison between experimental and simulated resistant torque $M_{b}$ + $M_{gear}$ for test 4 with a gear lubrication flow rate equal to 5 $\frac{L}{\min}$, see Table 3.

**Figure 44:**
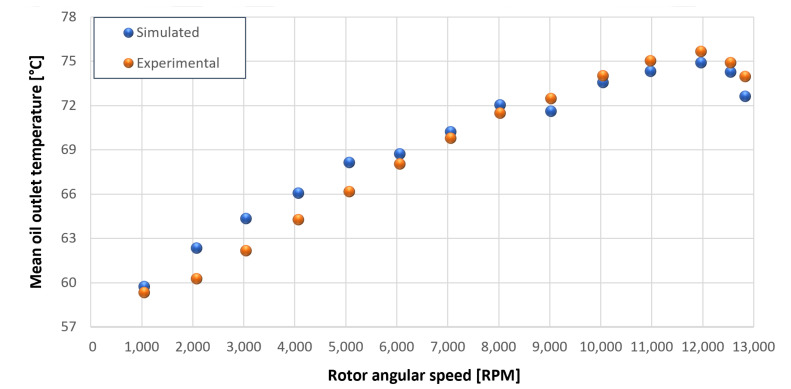
Comparison between experimental and simulated mean oil outlet temperature $T_{oil}$ for test 4 with a gear lubrication flow rate equal to 5 $\frac{L}{\min}$ for engine rotor, see Table 3.

**Figure 45:**
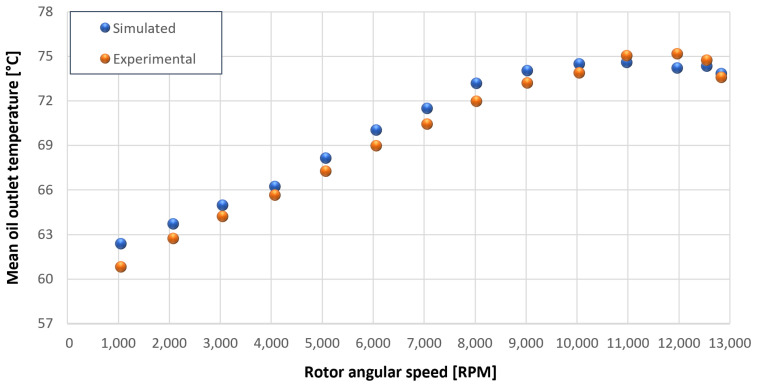
Comparison between experimental and simulated mean oil outlet temperature $T_{oil}$ for test 4 with a gear lubrication flow rate equal to 5 $\frac{L}{\min}$ for driven rotor, see Table 3.

**Figure 46:**
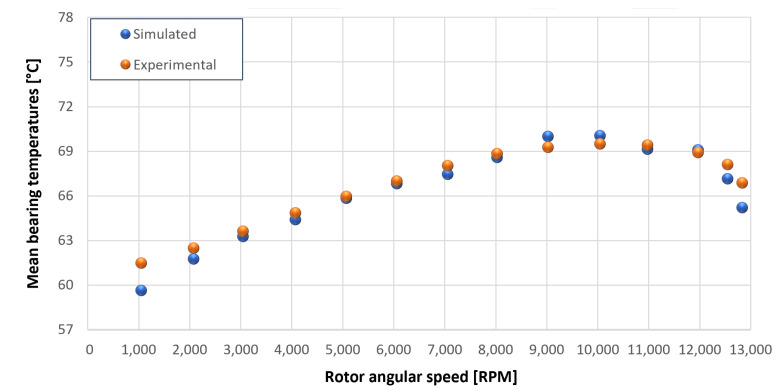
Comparison between experimental and simulated mean bearing temperatures $T_{oe}$ for test 4 with a gear lubrication flow rate equal to 5 $\frac{L}{\min}$ for engine rotor, see Table 3.

**Figure 47:**
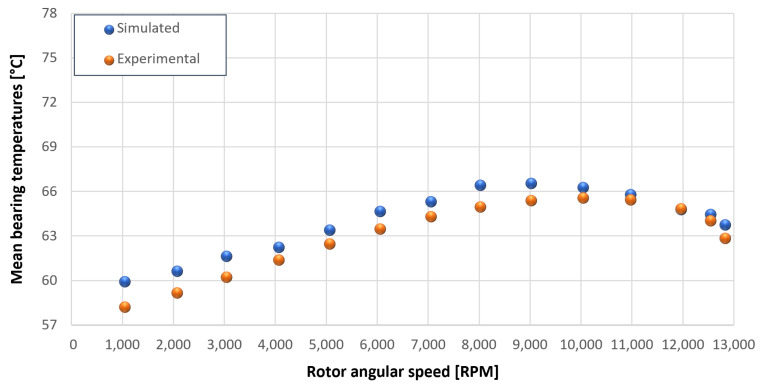
Comparison between experimental and simulated mean bearing temperatures $T_{oe}$ for test 4 with a gear lubrication flow rate equal to 5 $\frac{L}{\min}$ for driven rotor, see Table 3.

**Figure 48:**
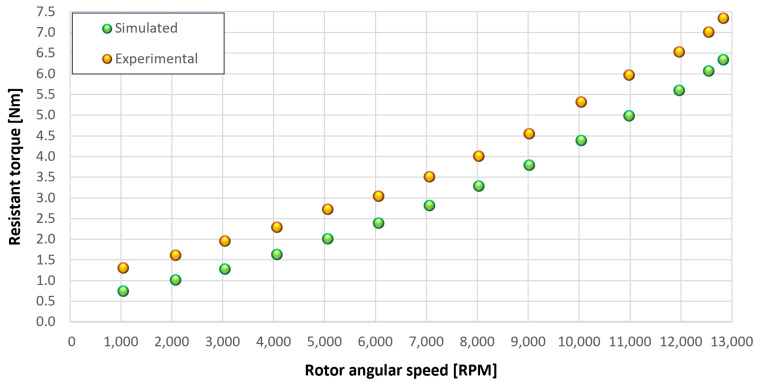
Comparison between experimental and simulated resistant torque $M_{b}$ + $M_{gear}$ for test 5 with a gear lubrication flow rate equal to 7 $\frac{L}{\min}$, see Table 3.

**Figure 49:**
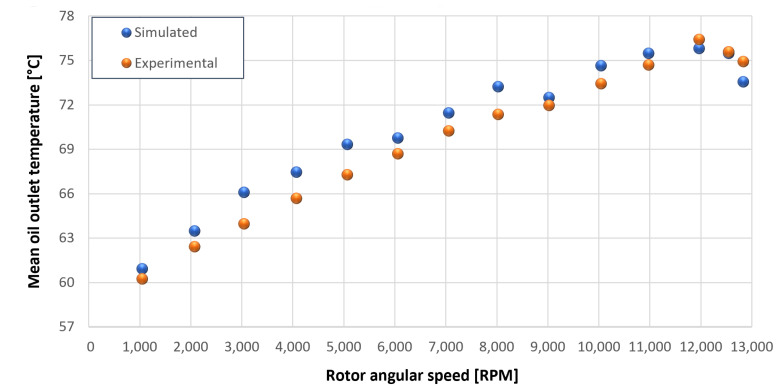
Comparison between experimental and simulated mean oil outlet temperature $T_{oil}$ for test 5 with a gear lubrication flow rate equal to 7 $\frac{L}{\min}$ for engine rotor, see Table 3.

**Figure 50:**
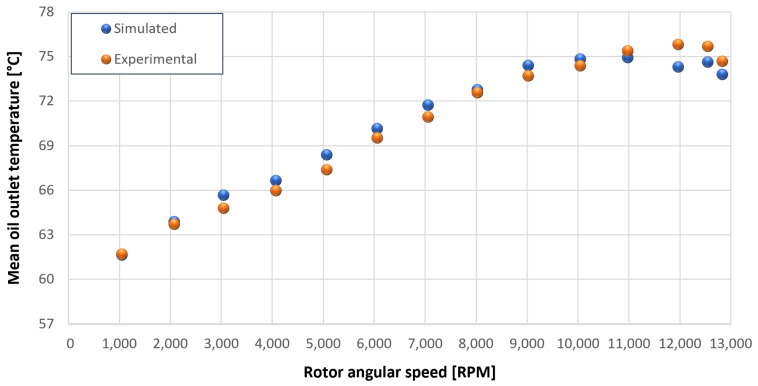
Comparison between experimental and simulated mean oil outlet temperature $T_{oil}$ for test 5 with a gear lubrication flow rate equal to 7 $\frac{L}{\min}$ for driven rotor, see Table 3.

**Figure 51:**
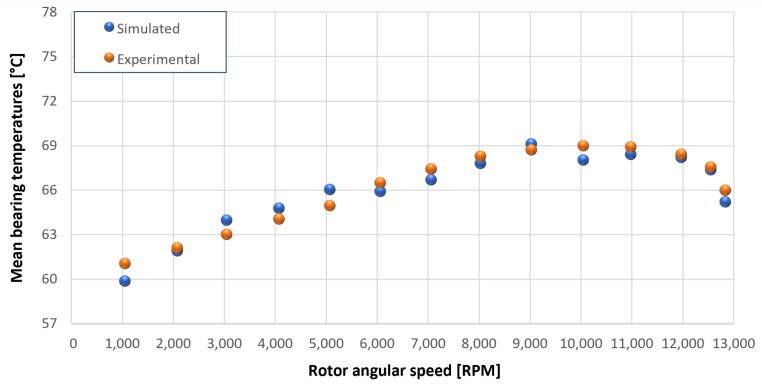
Comparison between experimental and simulated mean bearing temperatures $T_{oe}$ for test 5 with a gear lubrication flow rate equal to 7 $\frac{L}{\min}$ for engine rotor, see Table 3.

**Figure 52:**
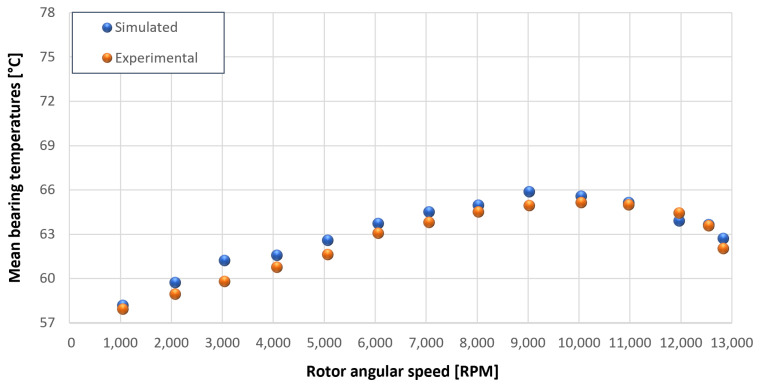
Comparison between experimental and simulated mean bearing temperatures $T_{oe}$ for test 5 with a gear lubrication flow rate equal to 7 $\frac{L}{\min}$ for driven rotor, see Table 3.

**Table 1:** Gears’ mechanical characteristics.

Teeth Num.	Module	Pitch Diam.	Width	Press. Angle
38 [-]	4 [mm]	152 [mm]	55 [mm]	20 [deg]

**Table 2:** Approximate values of the static and dynamic friction coefficients.

Surface State	Contact Materials	μ	$\mu_{a}$
Dry surface in air	Steel/PTFE	0.05	-
	Steel/Steel	0.11–0.40	0.60–0.80
	Steel/Nylon	0.15–0.40	-
	Steel/Bronze	0.30	0.35
	Steel-Ceramic	0.30–0.40	-
	Steel/Elastomers	1.60–10.0	-
	Steel/DLC	0.15	0.2
Limit lubrication	Metals/Metals	0.08–0.20	0.10–0.20
	Steel/Steel	0.07–0.16	0.08–0.20
	Steel/Bronze	0.10	0.15–0.20
	Steel/White metal	0.10	0.10
	Steel/DLC	0.10	0.15

**Table 3:** Experimental test plan.

Experimental Test Number [-]	Oil Initial Temperature Bearings [°C]	Oil Initial Temperature Gears [°C]	Lubricant Flow Rate Bearings [L/min]	Lubricant Flow Rate Gears [L/min]
Test 1	50	-	1.3	-
Test 2	50	20	1.3	1
Test 3	50	20	1.3	3
Test 4	50	20	1.3	5
Test 5	50	20	1.3	7

**Table 4:** Experimental test: data obtained by imposed experimental test data (see Table 3).

Experimental Test Number [-]	Test Chamber Temperature [°C]	Test Chamber Pressure [Pa]	Lubricant Oil Jet Temperature [°C]	Lubricant Oil Jet Pressure [bar]
Test 1	58.83	100,688.25	-	-
Test 2	56.29	101,736.77	28.58	2.06
Test 3	55.09	101,643.38	34.92	4.79
Test 4	58.03	101,628.43	44.27	8.46
Test 5	64.00	101,670.77	53.87	16.09

**Table 5:** Model parameter values.

Mechanical Parameter	Value
Bearing radial stiffness	$k_{b1}=4.45\times 10^{12}$ [N/m]
Bearing radial stiffness	$k_{b2}=1.25\times 10^{8}$ [N/m]
Bearing axial spring stiffness	$k_{ax}=10^{7}$ [N/m]
Bearing axial preload	$F_{ax}=2500$ [N]
Lubricant density	$\rho_{oil}=970$ [kg/m${}^{3}$]

**Table 6:** Numerical performances of the thermo-mechanical bearing model.

Mechanical Parameter	Value
Machines	HP Z620 [-]
CPU	Intel Xeon 2.60 GHz [-]
RAM	16 [GB]
Mean computational times	4.1 [seconds to simulate 1 s]

## Data Availability

Data supporting reported results can be found in the servers of the MDM Lab of the Department of Industrial Engineering of the University of Florence. Restrictions apply to the availability of these data.
